# Variability and Action Mechanism of a Family of Anticomplement Proteins in *Ixodes ricinus*

**DOI:** 10.1371/journal.pone.0001400

**Published:** 2008-01-02

**Authors:** Bernard Couvreur, Jérôme Beaufays, Cédric Charon, Kathia Lahaye, François Gensale, Valérie Denis, Benoît Charloteaux, Yves Decrem, Pierre-Paul Prévôt, Michel Brossard, Luc Vanhamme, Edmond Godfroid

**Affiliations:** 1 Laboratory for Molecular Biology of Ectoparasites, Institut de Biologie et de Médecine Moléculaires (IBMM), Université Libre de Bruxelles, Gosselies, Belgium; 2 Centre de Biophysique Moléculaire Numérique, Gembloux Agricultural University, Gembloux, Belgium; 3 Institute of Zoology, University of Neuchâtel, Neuchâtel, Switzerland; 4 Laboratory of Molecular Parasitology, Institut de Biologie et de Médecine Moléculaires (IBMM), Université Libre de Bruxelles, Gosselies, Belgium; Oregon Health & Science University, United States of America

## Abstract

**Background:**

Ticks are blood feeding arachnids that characteristically take a long blood meal. They must therefore counteract host defence mechanisms such as hemostasis, inflammation and the immune response. This is achieved by expressing batteries of salivary proteins coded by multigene families.

**Methodology/Principal Findings:**

We report the in-depth analysis of a tick multigene family and describe five new anticomplement proteins in *Ixodes ricinus*. Compared to previously described *Ixodes* anticomplement proteins, these segregated into a new phylogenetic group or subfamily. These proteins have a novel action mechanism as they specifically bind to properdin, leading to the inhibition of C3 convertase and the alternative complement pathway. An excess of non-synonymous over synonymous changes indicated that coding sequences had undergone diversifying selection. Diversification was not associated with structural, biochemical or functional diversity, adaptation to host species or stage specificity but rather to differences in antigenicity.

**Conclusions/Significance:**

Anticomplement proteins from *I. ricinus* are the first inhibitors that specifically target a positive regulator of complement, properdin. They may provide new tools for the investigation of role of properdin in physiological and pathophysiological mechanisms. They may also be useful in disorders affecting the alternative complement pathway. Looking for and detecting the different selection pressures involved will help in understanding the evolution of multigene families and hematophagy in arthropods.

## Introduction

Parasites probably affect every living organism and it may reasonably be estimated that at least half the animals on earth are parasites [1]. By definition, parasites live at the expense of their host but hosts defend themselves and, in turn, parasites evolve counter-measures. Parasitism is probably therefore a major driving force in evolution [1]. Bloodfeeding arthropods such as ticks constitute a very good example of the evolutionary arms race between hosts and parasites.

Ticks are obligate blood feeding arachnids. They infest many species of mammals, birds, reptiles and amphibians worldwide. They are the vectors of protozoan, bacterial and viral pathogens of prime medical and veterinary importance. Examples of such important pathogens are *Borrelia burgdorferi*, Tick-borne Encephalitis Virus (TBEV), *Babesia bovis* or *Theileria parva*, the respective agents of Lyme disease and viral encephalitis in humans, and babesiosis and theileriosis (East Coast Fever) in cattle [2]. Blood losses due to heavy infestation may weaken the animal, render it more susceptible to other diseases or cause death by exsanguination [3]. In addition, the occurrence of tick toxicosis including tick paralysis is associated with the presence of toxins in the saliva [4].

There are two main families of ticks, *Ixodidae* or hard ticks and *Argasidae* or soft ticks. The *Ixodidae* family is further divided into two subdivisions: Prostriata, which contains only the subfamily *Ixodinae*, and Metastriata, which includes the subfamilies *Bothriocrotinae*, *Amblyomminae*, *Haemaphysalinae* and *Rhipicephalinae* [3]. Argasid ticks typically feed for short periods of time (up to 2 hours) whereas Ixodid ticks remain attached to and feed on their vertebrate host for up to two weeks [3]. The feeding time of an adult *Ixodes ricinus* female is typically 7–10 days [3]. Such a long blood meal is only possible because these parasites have developed ways to circumvent host defense mechanisms including hemostasis (coagulation, platelet aggregation and vasoconstriction), the inflammatory response and innate and adaptive immunity [reviewed in 5], [6], [7], [8]. Furthermore, pain or itching caused by the inflammatory response stimulates hosts to scratch and dislodge the parasite.

The complement system is a first line of defence against invading pathogens and it links the innate and adaptive responses of the vertebrate immune system [reviewed in 9]. It consists of a cascade of plasma enzymes leading to activation of three effector mechanisms: (i) generation of the short potent pro-inflammatory peptides C3a and C5a, ii) deposition of opsonizing C3b proteins on cell surfaces, (iii) formation of the membrane attack complex (MAC). MACs create pores in the membrane, leading to cell death. Complement is activated *via* three main pathways. The classical pathway (CP) is initiated mainly when the C1 complex binds to the Fc region of certain antibody isotypes in immune complexes. The lectin-mediated pathway is activated by mannose-binding lectin interacting with mannose residues on microbial surfaces.

The alternative pathway (AP) is spontaneously activated by hydrolysis of plasma C3 into C3 (H_2_O). C3 (H_2_O) binds soluble factor B (fB). Bound fB is cleaved by serine protease factor D into soluble Ba peptide and the larger Bb fragment. The resulting C3 (H_2_O)Bb complex is the initial C3 convertase. It cleaves fluid-phase C3 into C3a peptide and metastable C3b. C3b binds covalently to a pathogen or cell surface via a short-lived thioester bond. Factor B interacts with C3b, leading to its cleavage by factor D and the formation of the C3 convertase (C3bBb). This complex generates new C3b molecules and amplifies the complement cascade by forming new C3 convertases or C5 convertases (C3b2Bb). C5 convertase cleaves C5 into C5a and C5b. C5b initiates the formation of MAC [9].

Host cells are protected from attack by the complement system by plasma and membrane-bound regulatory molecules that inactivate complement proteins. C3 convertases are deactivated by dissociation mediated by surface proteins such as Decay-Accelerating Factor (DAF) and Complement Receptor-1 (CR1), as well as soluble factor H. These proteins bind to C3b and displace Bb [9]. They also act as co-factors for serine protease factor I which cleaves C3b [10]. On the other hand, the half-life of C3 convertase is increased at least 10-fold by properdin [11]. It is present in the plasma in oligomer form (dimer, trimer or tetramer) [12], [13]. Each monomer is a 53 kDa protein composed of six repetitive thrombospondin domains (TSP), flanked with an N-terminal and C-terminal region [14], [15], [16]. Properdin binds to surface-bound C3b and increases its ability to interact with factor B [17]. It also binds to pre-formed C3 convertases leading to increased stability and preventing inactivation by regulators such as factor H and factor I [9]. Moreover, properdin oligomers attached to C3b on cell surfaces interact with preformed fluid-phase C3b or C3bBb through its other subunits [18]. The essential role of properdin in complement activation was demonstrated by the capacity of an anti-properdin monoclonal antibody to inhibit activation of the alternative pathway. This monoclonal antibody prevents the interaction between properdin and C3b [19].

The AP is the major line of defense against invading pathogens such as bacteria [20]. It is also involved in guinea pig resistance to the hard tick *Dermacentor andersoni* [21], [22]. Saliva or salivary gland extracts from *Ixodes dammini* [23], *I. hexagonus* and *I. uriae* [24], *I. scapularis* [25] and *I. ricinus* [24], [26] have been found to have an inhibitory activity on the alternative complement pathway. Valenzuela et al. [25] purified the active anticomplement component from the saliva of adult *I. scapularis*. N-terminal sequencing combined with the screening of a cDNA library led to the description of the coding sequence of a tick anticomplement protein named ISAC (*I. scapularis* anti-complement). Recombinant ISAC mimics the anticomplement activity of tick saliva. It interferes with the formation of C3 convertase from C3 and fB and destabilizes pre-formed C3 convertase. Sequences closely related to ISAC were then cloned by RT/PCR from *I. scapularis* nymphs [27], found by screening a cDNA library with sera from repeatedly infested guinea pigs [28] or by PCR-screening of a nymph cDNA library [29]. In *I. pacificus*, sequencing large numbers of cDNA clones from adult salivary glands led to the discovery of ISAC-I [30]. Finally, using degenerate primers designed from the published ISAC sequence, Daix et al. [31] recently cloned the related IRAC I and IRAC II from *I. ricinus*.

In soft ticks too, anticomplement activity is present in saliva and salivary gland extracts [32]. In *Ornithodoros moubata*, this activity is due to protein OmCI which inhibits both the alternative and classical pathways. Its sequence is unrelated to the *Ixodes* anticomplement molecules mentioned above (<15% amino-acid identity). OmCI binds to C5 component of the complement cascade and belongs to the lipocalin superfamily [33].

The recent characterization of large numbers of cDNA sequences from salivary glands of Ixodid ticks including *I. scapularis* [34], [35] and *I. pacificus* [30] indicated that most salivary proteins are expressed as large clusters of related proteins, probably coded by multigene families. Moreover, genome size and organization were examined in *Ixodes scapularis*, *Boophilus microplus* [36] and *Amblyomma americanum* [37]. These genomes are large: 2.1×10^9^, 7.1×10^9^ bp and 1.04×10^9^ bp respectively. Reassociation rates of genomic DNA indicate that they are composed mainly of moderately repetitive elements, which include transposable elements and members of multigene families. This organization in multigene families is therefore probably a major feature of hard tick genome organization and perhaps an adaptation to bloodfeeding.

In the work described here we completed an inventory of sequences related to *I. scapularis* anticomplement protein ISAC in the salivary glands of *I. ricinus*. We were able to detect five new sequences that we used to study diversification mechanisms possibly at work in a family of tick salivary proteins (hereby referred to as the IxAC family) and we investigated their action mechanism. The results showed that *I. ricinus* anticomplement proteins specifically bind to properdin, leading to the inhibition of the formation of C3 convertase and inhibition of the alternative complement activation pathway. Sequence diversification is associated with antigenic diversity rather than major divergence in molecular characteristics or activity, host specificity or stage specificity.

In this study, we investigated the general significance of multigene families in the context of a host-parasite relationship. This is a specific in-depth analysis of a tick multigene family. It highlights the value of performing dedicated gene-targeted inventories when studying specific aspects of tick adaptation to a bloodfeeding lifestyle. Remarkably, IxACs from *I. ricinus* are also the first inhibitors that specifically target a positive regulator of complement.

## Results

### A large family of anticomplement proteins in the hard tick *Ixodes ricinus*

The fragments of two genes related to the prototypical *Ixodes scapularis* anticomplement protein ISAC of Valenzuela et al. [25] were found by serendipity in cDNA from pooled salivary glands. The complete coding sequences as well as parts of the 3′ and 5′ UTR's were then reconstituted by RACE. The new genes (accession numbers: AM407396 and AM407397) coded for two new proteins showing ∼40 % identity with ISAC and the recently described IRAC I and IRAC II from *I. ricinus* [31], but over 65 % identity with each other. This led us to suspect the existence of a much larger family of anticomplement proteins in *I. ricinus*.

In order to make as complete an inventory of this family as possible, a total of 2 different reverse transcription experiments, 6 different PCR amplifications and 12 ligations were performed on polyA+ RNA from salivary glands of *I. ricinus* females (Table S1). 122 clones with inserts of the expected size (≥600 bp) were sequenced. A few were disregarded as they coded for proteins unrelated to anticomplement proteins (e.g. ribosomal proteins). A few additional clones with inserts smaller than expected (400–500 bp) were sequenced too. They were found to code for homologs of uncharacterized “putative salivary proteins” from *I. scapularis* and *I. pacificus*. Of the 118 anticomplement-like clones found, most could be assigned to previously described IRAC I, IRAC II, AM407396 and AM407397 on the basis of sequence identity. AM407396 was the most frequent (46.6 %) followed by AM407397 (23.3 %), IRAC I (15.5 %) and IRAC II (0.9%). Three additional new sequences were also identified. They were assigned accession numbers AM407398, AM407399 and AM407400 respectively. They accounted for 3.4 %, 8.6 % and 1.7% of clones, respectively. Sequences found to be identical in ≥3 independent clones were considered genuine. AM407400 was only represented by two clones which showed a difference of two nucleotides. Therefore, the latter sequence was confirmed independently by amplifying internal fragments with gene-specific primers from salivary gland cDNA. Finally, the same set of sequences was found from the various independent RT-PCR experiments (Table S1). Overall, the results suggested that a complete or near-complete inventory of IxAC anticomplement messengers from the *Ixodes ricinus* salivary gland was achieved at least for the population investigated.

PCR experiments using the primers listed in Table 1 were also performed on cDNA from pooled salivary glands of 3 day-fed male and female *R. appendiculatus*. No PCR products were observed (not shown). We were nevertheless able to amplify the coding sequence for the known lipocalin RaHBP-2 from the same cDNAs using specific primers. Interrogation of databases including the preliminary releases of sequences from the non-*Ixodes* hard tick *R. appendiculatus, B. microplus, A. americanum and A. variegatum* genome projects yielded no or only insignificant hits.

**Table 1:** PCR primers for RT-PCR inventory of *I. ricinus* genes coding for anticomplement proteins.

Primer	sequence	Tm (°C)	Designed from	Usage
IRI (fw)	5′-ACCATGARGACTGYGCTGACCTGTGC –3′	70–74°c	5′ end of *I. ricinus* anticomplement CDS	Inventory in adult SG
IXO (fw)	5′-ACCATGARGACTGYGYTBACCTGTGC –3′	66–74°c	5′ end of *Ixodes* spp. anticomplement CDS	
Not1 primer (rev)	5′-AGAATTCGCGGCCGCAGGAAT -3′	66°c	3′ race primer for *Not1*Oligod(T)18 RT primer	
Generacer 3′	5′–GCTGTCAACGATACGCTACGTAACG-3′	76°c	3′ race primer for Generacer Oligo dT primer	
UTR1 (rev)	5′-CACCACGCAGTGCCATCTGT–3′	64°c	3′ UTR of IxAC-B1 to B5	
UTR2 (rev)	5′–ATGGGTATCGGCATACCGATC-3′	64°c	3′ UTR of IxAC-B1 to B7	
UTR3 (rev)	5′–GTTTCTGGTAATAACCGGGTG–3′	62°c	3′ UTR of IRAC-I and IRAC-II	Inventory in nymphs and larvae
UTR4 (rev)	5′–CGYATCAGAACTRTGCTTGCAC–3′	64–68°c	3′ UTR of Isac-like	
CDSrev1 (rev)	5′–TCAKGSGATGGCCTCARGTTC–3′	64–68°c	C-terminal end of IRACI and II CDS	
CDSrev2 (rev)	5′–YTYRRASRGGGTGGTCGG–3′	54–64°c	C-terminal end of IxAC-B1 to B5 CDS	

Forward primers IRI and IXO were designed manually from the 5′ end of coding sequences of anticomplement proteins available at the start of this project from *I. ricinus* and *Ixodes* spp. respectively. The trinucleotide ACC was added 5′to the start codon to improve eukaryotic expression. Commercial reverse primers Generacer 3′ and Not1 are available from Invitrogen and Amersham Biosciences, respectively. Family-specific reverse primers were designed from UTR or coding sequences of the different subfamilies of tick anticomplement proteins as indicated. Calculated mean melting temperatures (Tm) are also indicated. CDS, coding sequences; UTR, untranslated region; SG, salivary gland.

### Phylogenetic analysis of tick anticomplement sequences

Nucleotide and peptide sequences of ISAC, IRAC I and AM407396 were used to interrogate databases with the same results. A total of 48 entries were recovered (Table S2). These were from *I. scapularis* (45 entries), *I. ricinus* (2 entries) and *I. pacificus* (1 entry). They had been cloned from salivary glands (30), whole fed nymphs and nymph salivary glands (15) or unspecified tick material (3).

**Figure 1:**
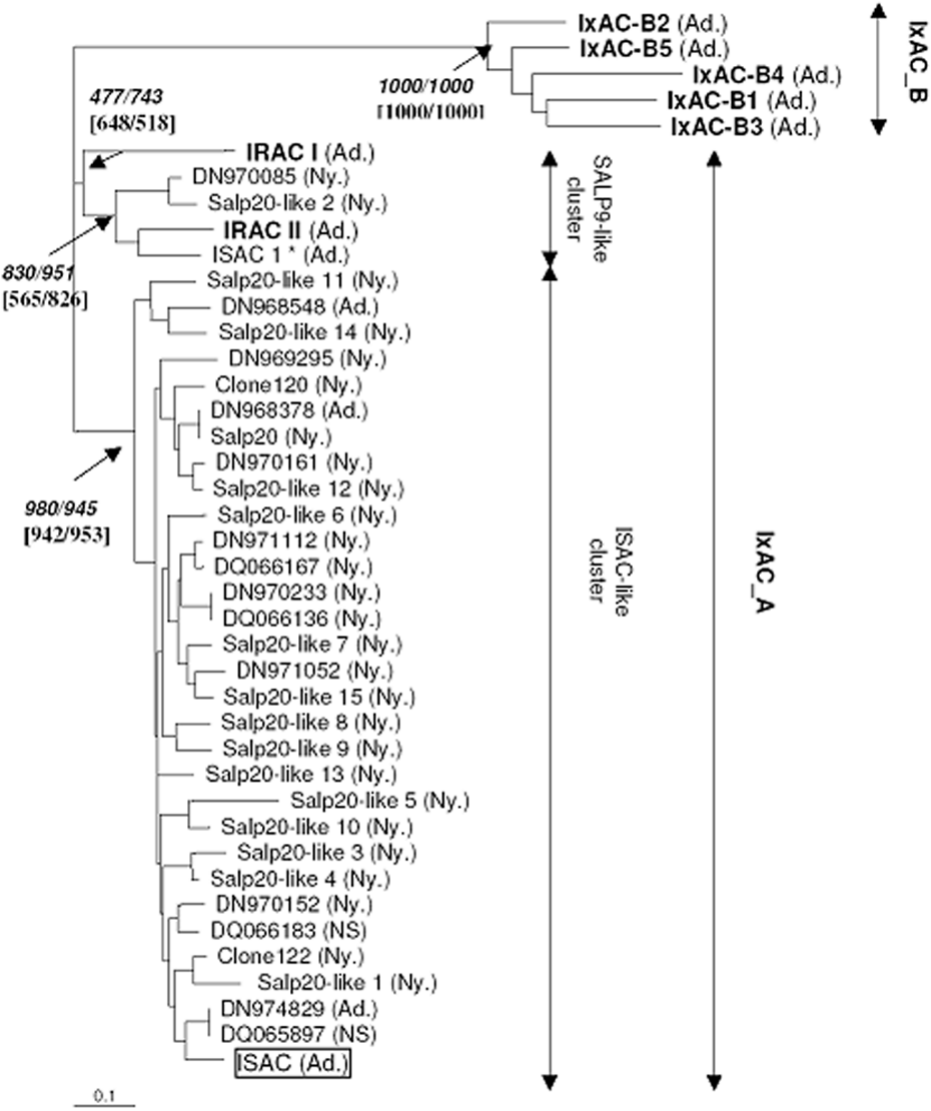
Phylogenetic analysis of *Ixodes* anticomplement proteins. A distance dendrogram was constructed from an alignment of 41 tick mature anticomplement proteins using programs in the Phylip 3.65 package (see text). Branch length is proportional to distances between peptide sequences. The bootstrap values are indicated near major nodes, calculated from 1000 replicates of the peptide and nucleotide sequence alignments, respectively. Bold characters: *I. ricinus* entries; *: *I. pacificus* sequence; all others are from *I. scapularis*. Prototypical ISAC is boxed. Sequences are identified by their accession number in databases or by descriptive names when available. Ad., isolated from adults; Ny., isolated from nymphs; NS, not specified.

Two sequences containing ambiguous positions and ten sequences with incomplete coding sequences for the mature protein, including Salp9 (AF278574) and Isac-like clone 113 (AY956386), were initially discarded. These 12 entries were from *I. scapularis*. The remaining 36 entries were aligned with our five new sequences from *I. ricinus* (AM407396 to AM407400). Distance dendrograms were constructed from alignments of nucleotide sequences or predicted amino-acid sequences of putative mature proteins translated from the open reading frames (Figure 1). All sequences clustered into two main groups or subfamilies, IxAC-A and IxAC-B, which were strongly supported by bootstrap analysis (1000/1000). IxAC-A could be further divided into two clusters. A first, large, group contained only *I. scapularis* sequences closely related to prototypical ISAC. It was strongly supported by bootstrap analysis (>900/1000). A second, smaller, cluster contained Isac-1 from *I. pacificus*, IRAC II from *I. ricinus*, Salp20-like protein 2 and EST n° DN970085 from *I. scapularis*. Bootstrap support was lower (≥830/1000). IRAC I could not be joined robustly (bootstrap value <750/1000) to any of the previous two clusters. The IxAC-B subfamily contained our five new sequences AM407396 to AM407400 from *I. ricinus* but none from other tick species. No robust cluster emerged within this subfamily.

The maximum-likelihood method was also applied to the initial nucleotide and amino-acid alignment of putative mature proteins. It supported the same topology as the distance method with slightly different bootstrap values (Figure 1). The two subfamilies were strongly supported (1000/1000). The two clusters within IxAC-A were also recovered but bootstrap support was lower than with the distance method. Again, IRAC I could not be placed robustly in any of the two clusters within IxAC-A (bootstrap support <650/1000).

The overall topology of the distance trees was not altered after including the leader peptide sequences in the alignments (not shown) or the 12 discarded sequences (not shown). Most of the latter clustered with ISAC within the ISAC-like cluster (not shown). Only Salp9 grouped with Isac-I, IRAC II and EST n° DN970085 within the second cluster in IxAC-A. Salp9, a 79 residues peptide, aligned to the C-terminal half of DN970085 to which it showed 90% identity. Because ISAC and Salp9 were the earliest tick anticomplement sequences published, we decided to name the large and small clusters within IxAC-A “ISAC-like” and “Salp9-like”, respectively.

We therefore decided to rename the five new sequences (AM407396 to AM407400) IxAC-B1 to IxAC-B5 to indicate the fact that they clustered into the new group or subfamily IxAC-B and away from the previously described IRAC I and IRAC II which belong to the IxAC-A subfamily.

**Figure 2:**
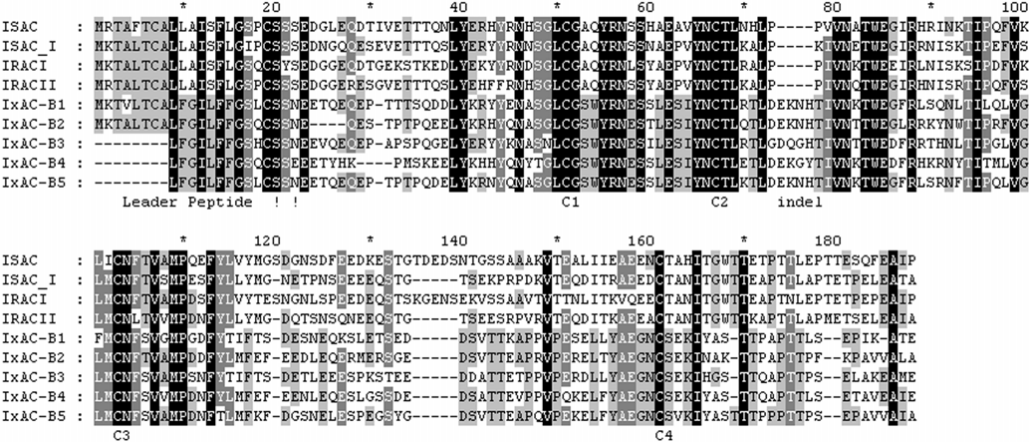
Alignment of *Ixodes* anticomplement proteins. The 7 anticomplement proteins from *I. ricinus* (IRAC I and II; IxAC-B1 to B5) were aligned with the prototypical anticomplement protein from *I. scapularis* (ISAC) and the homolog from *I. pacificus* (ISAC I). Individual residues printed in white on a black background are conserved in all 9 aligned sequences; white residues on a grey background are conserved in 7 or 8 of 9 entries; black residues on a grey background are conserved in 5 or 6 entries; black residues on a white background are conserved in less than 5 entries. -, gap; ! !, region of predicted signal peptide cleavage; C1 to C4, conserved cysteine residues.

Percentages of identity and similarity were calculated for representative IxACs (Table 2). Within a subfamily, amino-acid sequences were over 60 % identical whereas identity dropped to ∼40 % between the two subfamilies. The two subfamilies could also be differentiated by an indel of 4 amino-acids (position 74 to 77 in Figure 2). Within the Isac-like cluster or within the Salp9-like cluster, amino-acid sequences were at least 70% identical.

**Table 2:** Nucleotide and amino-acid identity/similarity of mature anticomplement proteins.

	Isac	Isac-1	IRAC I	IRAC II	IxAC-B1	IxAC-B2	IxAC-B3	IxAC-B4	IxAC-B5
Isac		61/76	63/75	62/77	37/53	39/55	38/52	36/51	40/54
Isac-1	76		62/74	78/90	40/57	44/58	40/55	37/55	40/58
IRAC I	78	79		61/74	35/52	38/53	37/52	37/50	36/52
IRAC II	76	91	78		37/57	41/58	37/55	37/57	41/58
IxAC-B1	51	54	51	53		66/77	68/81	64/78	75/82
IxAC-B2	51	55	50	53	79		63/76	66/77	73/83
IxAC-B3	51	54	51	53	81	79		63/76	65/76
IxAC-B4	49	53	49	52	78	79	77		68/76
IxAC-B5	52	54	50	53	86	82	80	81	

Percent identity/similarity of amino acid sequences are indicated in the upper right triangle. Percent identities of nucleotide sequences are indicated in the lower left triangle.

According to information provided on the entry files or in the original publications, the sequences had been obtained from adults (12), nymphs (34) or unspecified stages (2). However, no robust “adult-only” or “nymph-only” clusters could be discerned (Figure 1). Moreover, “adult” EST n° DN968378 was found to be 100% identical to “nymphal” Salp20 (AF209917) (Figure 1).

To summarize, phylogenetic analysis of all available tick anticomplement sequences indicated that they robustly segregated into two distinct groups or subfamilies, which we termed IxAC-A and IxAC-B. Within-group amino-acid identity was >60% whereas between-group identity dropped to ∼40%. The larger IxAC-A contained sequences from *I. scapularis*, *I. ricinus* and *I. pacificus*. It could be subdivided into two or possibly three clusters. Our new sequences from *I. ricinus* constituted a completely new group which we named IxAC-B. No stage-specific group of sequences was identified at this point of the research.

### Protein properties

The properties of these newly discovered proteins were predicted from their amino-acid sequences and compared to prototypical ISAC from *I. scapularis* and related Isac-1 from *I. pacificus*. Calculated PM and pI ranged from 17.46 to 18.03 and 4.01 to 4.29 respectively (Table 3).

**Table 3:** Calculated properties of anticomplement proteins.

Sequence name	Accession number	Precursor size (aa)	Signal peptide cleavage site		Mature^c^ MW (kDa)	Mature^c^ pI	N-gly.	O-gly.
			Most likely	Less likely				
ISAC^a^	AAF81253.1	184	21–22	22–23	18.14	4.19	6	12
Isac-1^b^	AAT92205.1	178	22–23	21–22	17.55	3.99	5	15
IRAC I	CAD82867	184	19–20	22–23	18.03	4.01	5	8
IRAC II	CAD82868	178	21–22	22–23	17.46	4.29	5	12
IxAC-B1	AM407396	177	21–22	19–20, 20–21	17.58	4.00	6	12
IxAC-B2	AM407397	174	19–20	21–22	17.69	4.40	5	8
IxAC-B3	AM407398	178	19–20	21–22	17.85	4.01	5	12
IxAC-B4	AM407399	175	21–22	19–20	17.88	4.14	7	7
IxAC-B5	AM407400	179	21–22	19–20, 20–21	17.66	4.15	7	11

Amino-acid sequences were deduced from the sequenced open reading frames. Indicated values were calculated from deduced amino-acid sequences using online programs at CBS and EBI (see text). Signal peptide cleavage sites are indicated by the position of the residues between which the cleavages were predicted to occur. (a) *I. scapularis* sequence [25]. (b) *I. pacificus* sequence [30]. (c) after removal of the predicted signal peptide at the most likely cleavage position (see text). aa, amino-acids; MW, molecular weight; kDa, kiloDaltons; pI, isoelectric point; N-gly., number of predicted N-glycosylation sites; O-gly., number of predicted O-glycosylation sites.

All anticomplement proteins presented four conserved cysteine residues predicted to make two disulphide bridges (Figure 2).

Most likely signal peptide cleavage sites for representative IxACs are indicated in Table 3 and Figure 2. In each individual peptide sequence, SignalP predicted a second or even a third probable, though less likely, cleavage site. These were at position 19 (after C residue), 21 (after SS residues) or 22 (after SSN/E). Altogether, cleavage at any 3 locations within the C↑SS↑(S) ↑E/N motif is theoretically possible.

The presence of a signal peptide and the absence of any hydrophobic transmembrane region suggests that these proteins are secreted. This was supported by a TargetP program analysis and confirmed experimentally as recombinant IxACs were recovered in the culture medium after transfection of COS7 or 293T cells.

**Figure 3:**
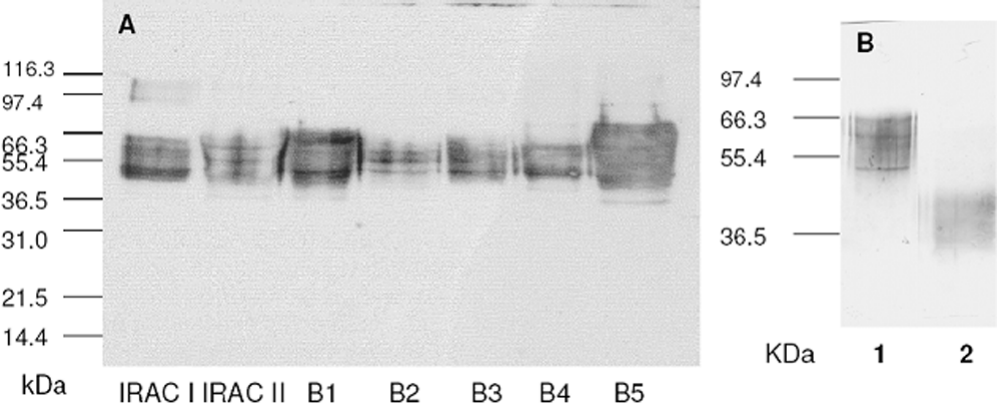
Western blot analysis of recombinant IxAC-V5His proteins from *I. ricinus*. Standardised amounts of recombinant IxAC-V5His proteins from supernatants of transfected 293T cells were analysed by SDS/PAGE and detected by western blotting using an anti-V5 monoclonal antibody. A) Parallel analysis of IxACs, B) N-deglycosylation of IxAC-B1-V5His.1, untreated, 2, incubated with PNGase (New England Biolabs).

In a western blot analysis using an anti-V5 antibody, all recombinant IxACs from *I. ricinus* appeared as a series of thin bands at 50–70 kDa (Figure 3a). The apparent molecular weights are consistent with reported values for purified native anticomplement proteins from *I. scapularis* (∼65 kDa) [25] and *I. damini* (∼49 kDa) [23]. They contrast with the predicted MW of ∼18 kDa. This difference and the appearance of the bands were in agreement with extensive glycosylation. Indeed, several consensus sites for N- and O- glycosylation were found in the sequences (Table 3). Furthermore, the presence of N-linked glycosylation was experimentally confirmed by treatment with N-glycosidase F leading to a fall in observed MW to 35–45 kDa (Figure 3b). Recombinant Salp20 expressed in insect cells also appears as a smear possibly representing differentially glycosylated forms of the protein [29]. The authors experimentally confirmed the presence of N-linked and O-linked sugars.

**Figure 4:**
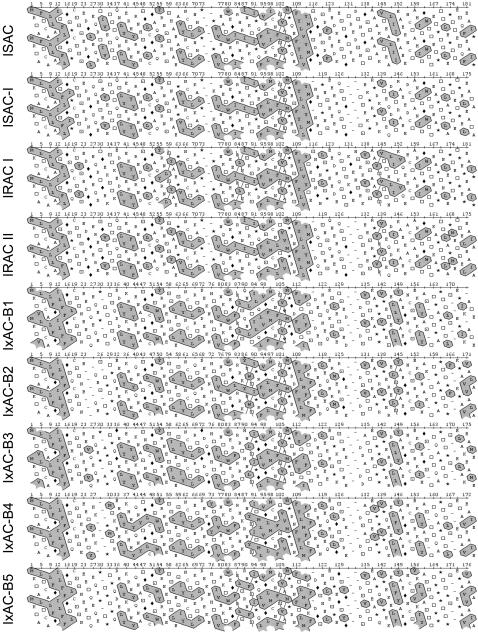
Comparison of *Ixodes* anticomplement protein tertiary structure. Aligned IxAC amino-acid sequences from *I. ricinus* were submitted to hydrophobic cluster analysis (HCA). Groups of adjacent hydrophobic residues are outlined and shaded. Proline (asterisk), glycine (open rectangle), serine (dotted square) and threonine (open square) are highlighted. The overall distribution of hydrophobic clusters and their size, shape and orientation are very similar.

Finally, hydrophobic cluster analysis (HCA) showed that the distribution of clusters of hydrophobic amino-acids had a nearly identical distribution in all 7 IxACs from *I. ricinus* as well as in ISAC and ISAC-1 (Figure 4). This suggested that these 9 proteins had identical folds or tertiary structures.

### The excess of non-synonymous over synonymous changes indicates that the coding sequences had undergone diversifying selection

Calculation of the percentage identity between 7 *I. ricinus* anticomplement sequences indicated that they were more closely related at the nucleotide level than at the amino-acid level (Table 2).

A theoretical ancestral sequence was also re-constructed using the Ancescon program. It was aligned to the 7 actual sequences. The numbers of synonymous changes per synonymous sites (dS) and non-synonymous changes per non-synonymous sites (dN) were calculated using the Nei-Gojobori method. Values for dN/dS were consistently >1 for pairwise comparisons of actual sequences with one another and with the putative ancestral sequence (Table 4, higher-right triangle). The ratio from overall means of dN and dS values was 2.44. Fisher's exact test for positive selection did not reject the hypothesis of dN >dS except in the case of IRAC I compared to IRAC II (P value <0.05) (Table 4, lower left triangle).

**Table 4:** Evidence for positive selection in *I. ricinus* IxAC coding sequences.

	Irac I	Irac II	Ancestral	IxAC-B1	IxAC-B2	IxAC-B3	IxAC-B4	IxAC-B5
Irac I		5.92	2.06	2.30	2.18	2.30	2.41	2.08
Irac II	0.028		1.77	2.38	2.03	2.30	2.38	2.05
Ancestral	1.000	1.000		2.82	1.92	2.54	2.46	2.07
IxAC-B1	1.000	1.000	1.000		4.13	2.63	3.39	3.28
IxAC-B2	1.000	1.000	1.000	0.223		3.63	3.28	3.17
IxAC-B3	1.000	1.000	1.000	1.000	0.435		3.25	3.57
IxAC-B4	1.000	1.000	1.000	1.000	1.000	1.000		2.51
IxAC-B5	1.000	1.000	1.000	0.491	1.000	0.441	1.000	

Pairwise dN and dS values were calculated using the Nei-Gojobori method as implemented in the Mega3 package. dN/dS ratios are indicated in the upper right triangle. Fisher's test P values are indicated in the lower left triangle. A putative ancestral coding sequence for *I. ricinus* anticomplement proteins was inferred from an alignment of the IxAC from *I. ricinus* coding sequences using the Ancescon package.

All the data therefore show that diversifying selection had taken place within the IxAC family in *I. ricinus*.

### IxACs from *I. ricinus* inhibit the alternative complement pathway (AP) but not the classical pathway (CP)

**Figure 5:**
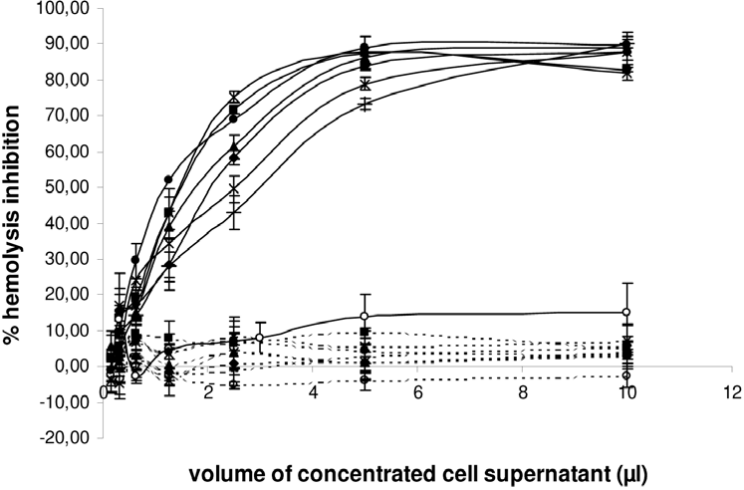
Effect of recombinant *I. ricinus* IxAC proteins on the alternative and classical pathways of complement activation. Assays of the alternative (AP, solid lines) and classical (CP, dashed lines) complement activation pathway were conducted in the presence of normalized amounts of recombinant *I. ricinus* IxACs produced in the supernatant of transfected 293T cells. Values for the percent inhibition of rabbit red blood cell lysis in the presence of human serum are indicated. The values are means ± standard deviation of triplicates. RaHBP2 was used as negative control. Black diamond :IRAC I; black square:IRAC II; black triangle:IxAC-B1; cross:IxAC-B2; star:IxAC-B3; closed circle:IxAC-B4; Plus:IxAC-B5; open circle:RaHBP2.

The effect of similar amounts of the seven *I. ricinus* IxACs transiently expressed in 293T cells (Figure 3) were assessed in hemolytic assays of both the classical (CP) and alternative complement pathways (AP). A clear dose-dependent inhibition of the AP was observed for all seven recombinant proteins tested as they inhibited the lysis of rabbit erythrocytes by normal human serum. The shape of the curves and plateau values for hemolysis inhibition were identical for the seven proteins (Figure 5).

Addition of recombinant IxACs in the AP assay 15 minutes after adding red blood cells to human serum in the AP assay showed that they were able to inhibit ongoing hemolysis of rabbit erythrocytes. (Figure S1).

The same dilutions of the 7 recombinant proteins were retested in the CP assay in the presence of normal human serum. As shown in Figure 5, there was no inhibition of lysis of antibody-sensitized sheep erythrocytes.

In both assays, no inhibition was observed after addition of recombinant RaHBP2.

We therefore concluded that the 7 IxAC from *I. ricinus* all had a similar inhibitory effect on the alternative but not the classical complement activation pathway.

### IxACs inhibit the cleavage of human C3 and factor B

During complement activation by the alternative pathway, plasma protein C3 is cleaved into the large opsonizing factor C3b and the small pro-inflammatory peptide C3a and precursor B is cleaved into a large Bb fragment and a small Ba peptide. We investigated the effect of *I. ricinus* IxAC proteins on the cleavage of factor B and on the production of C3a in the AP assay. Supernatants of completed AP hemolytic assays were analyzed by Western blot using antisera to fB or C3a.

**Figure 6:**
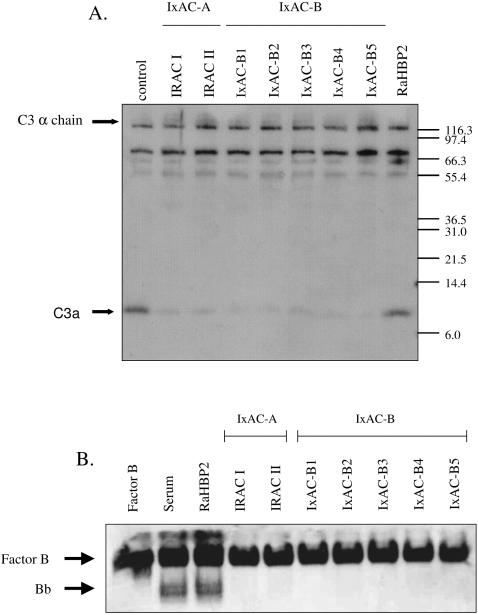
Inhibition of C3a formation and factor B cleavage. Aliquots of supernatant from AP hemolysis assays conducted in the presence of standardized amounts of IxACs from *I. ricinus* and unrelated control RaHBP2 were analyzed by western blotting. Panel A: Blots from gels run under denaturing conditions were probed with monospecific anti-C3a serum. The α-chain of precursor C3 (116 kDa) and the C3a peptide (∼10 kDa) are indicated by arrows. Panel B: Blots from gels run under non-denaturing conditions were probed with a antiserum to factor B. Purified factor B was used as a positive control.

As shown in Figure 6A, anti-C3a antibody recognized major bands at 116 kDa, 77kDa and ∼10 kDa. Arrows indicate clearly identifiable bands; they corresponded to the α-chain of C3 (115 kDa) and the small C3a peptide (9 kDa) [38], [39]. The latter was almost completely suppressed in samples from assays run in the presence of recombinant IxACs as compared to sample from assays run in the presence of RaHBP2 or without added protein.

Antiserum to factor B recognized purified factor B as a single band on a non-denaturating western blot (Figure 6B). A second band was recognized in samples from control AP assays run in the absence of added protein or in the presence of unrelated RaHBP2. It resolved into two distinct bands presumably corresponding to differently charged forms of Bb [39], [40]. It was absent in a sample from AP assays run in the presence of recombinant IxACs.

We concluded that *I. ricinus* IxAC inhibited the formation of C3a and cleavage of fB. Moreover, there were no detectable differences in the degree of this inhibition between different members of the two IxAC sub-families.

### *I. ricinus* IxACs specifically interact with properdin

**Figure 7:**
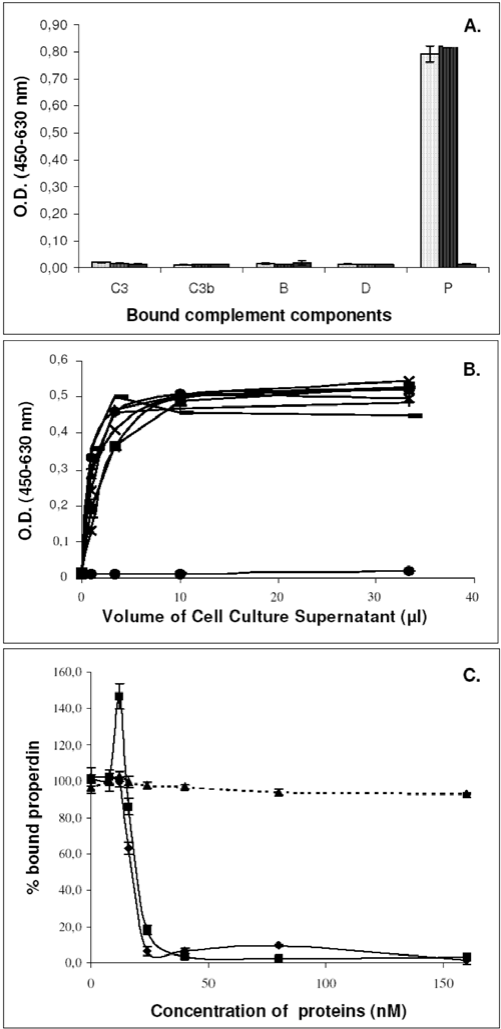
ELISA analysis of the binding of IxAC proteins to immobilized C3 convertase components. Panel A: Binding of IxACs to AP components. Purified recombinant IRAC II, IxAC-B1 or unrelated protein Iris were added to microtiter wells previously coated with purified factors C3, C3b, fB, fD or properdin (P). Bound proteins were detected with an anti-V5 monoclonal antibody using an ELISA format. Light dotted histogram: IRAC II; dark dotted histogram: IxAC-B1; black histogram: Iris. Panel B: Increasing amounts of normalized supernatant from transfected culture 293T cells were added to immobilized properdin. Bound IxACs were detected with an anti-V5 antibody. Black diamond: Iris; black square: IRAC I; black triangle: IRAC II; cross: IxAC-B1; star: IxAC-B2; closed circle: IxAC-B3; plus: IxAC-B4; minus : IxAC-B5. Panel C. Competition between properdin and IxACs for C3b binding. Purified properdin and increasing amounts of IRAC II, IxAC-B1 or unrelated control IRIS were added simultaneously to C3b-precoated microtiter wells. Bound properdin was detected with an anti-properdin monoclonal antibody. Black diamond: IRAC II; black square: IxAC-B1; black triangle: Iris.

We then attempted to identify the target(s) of IxACs using ELISA methodology. Components of C3 convertase (i.e. C3, C3b, fB, fD or properdin) were coated on microtiter plates and incubated with recombinant IxAC_V5His. Binding of IxACs was monitored using an anti-V5 antibody. We first tested one member of the IxAC-A subfamily and one from the IxAC-B subfamily. IRAC II and IxAC-B1 purified from the baculovirus/Sf9 expression system, but not unrelated protein Iris, strongly bound to properdin. They did not bind to C3, C3b, fB or fD (Figure 7A). In addition, standardized amounts of the seven *I. ricinus* IxACs but not RaHBP2 bound to properdin in a dose-dependent manner (Figure 7B).

We also tested the binding of properdin to C3b-coated plates in the presence of increasing amounts of IRAC II, IxAC-B1 and control Iris. Binding was revealed by a monoclonal antibody to properdin (Figure 7C). Increasing amounts of IRAC II, IxAC-B1 but not Iris lead to a decrease in the amount of bound properdin. We concluded that *I. ricinus* IxACs specifically interacted with properdin and prevented its binding to C3b. Again, no difference could be discerned amongst IxACs.

### IxAC proteins inhibit the formation of the C3 convertase complex by interacting with properdin

**Figure 8:**
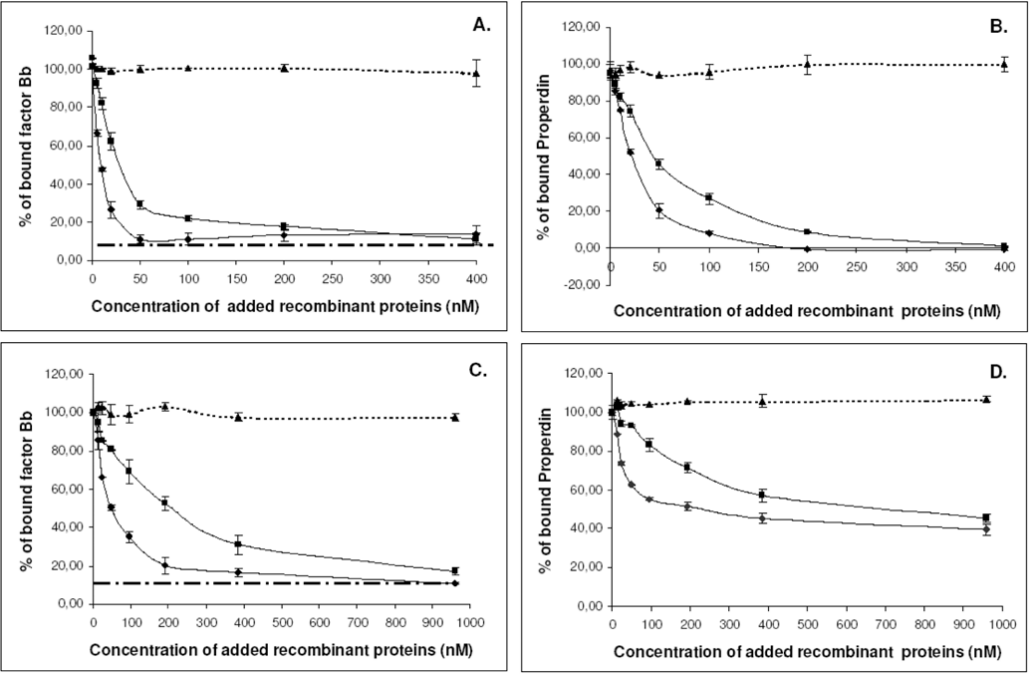
Effect of IxAC proteins on the formation and stability of C3 convertase. Panel A and B: The effect of IxAC proteins on the formation of C3 convertase was evaluated by incubating simultaneously purified factors B, D and properdin with increasing amounts of IRAC II or IxAC-B1 on C3b-coated wells. Panel C and D: The effect of IxAC proteins on the stability of C3 convertase was assessed by incubating preformed C3 convertase (fB, fD and properdin pre-incubated for 1 hour) on C3b-coated plates with increasing amounts of recombinant IxACs. Bound factor B or properdin was detected with an anti-factor B antibody (A–C) or anti-properdin antibody (B–D), respectively. Recombinant IRIS was used as negative control. Black diamond: IRAC II; black square: IxAC-B1; black triangle: IRIS.

We also studied the effect of *I. ricinus* IxACs on the formation and stability of the alternative pathway C3 convertase (C3bBbP). This was reconstituted *in vitro* by adding purified components fB, fD and properdin to C3b-coated plates. Bound Bb and properdin were detected using specific antibodies. Approximately 10 times less bound Bb was detected in the absence of properdin than in its presence (Figure 8A). We tested the effect of one member of each of the two IxAC subfamilies on the formation and stability of this complex.

In a first series of experiments, increasing amounts of purified IRAC II and IxAC-B1 were added together with the individual convertase components to C3b-coated plates. We observed a dose-dependent decrease in the amount of bound Bb (Figure 8A) or properdin (Figure 8B), indicating inhibition of complex formation. At the highest protein concentrations (≥200 mM), the amount of bound Bb dropped to values observed when reconstituting the C3 convertase without properdin (Figure 8A). No such effect was observed with the unrelated Iris protein (Figure 8).

In a second series of experiments, C3bBbP was pre-formed on ELISA plates and then incubated with increasing amounts of IRAC II and IxAC-B1 proteins and the unrelated control Iris protein (Figure 8C and D). The results indicated that IxAC proteins induced the displacement of all pre-bound factor Bb (Figure 8C) and about 50 % of pre-bound properdin.

We also performed time-course experiments of C3 convertase formation with (C3bBbP) or without properdin (C3bBb) in the presence of 200 mM IRAC II, IxAC-B1 or Iris. The amount of bound Bb was much lower in the absence of properdin than in its presence. In this case, the presence of IxACs or Iris had no effect, indicating that the proteins had no direct effect on the interaction between C3b and Bb. On the contrary, the formation of C3 convertase in the presence of properdin was strongly affected by IxACs. Values of bound Bb dropped to values observed without properdin (Figure S2).

Overall, these results show that *I. ricinus* IxAC proteins inhibit the formation of the C3 convertase complex by interacting specifically with properdin. They also induce the displacement of pre-bound properdin, and indirectly, Bb, in a dose-dependent manner.

### IxAC proteins inhibit complement activation on agarose-coated ELISA plates

**Figure 9:**
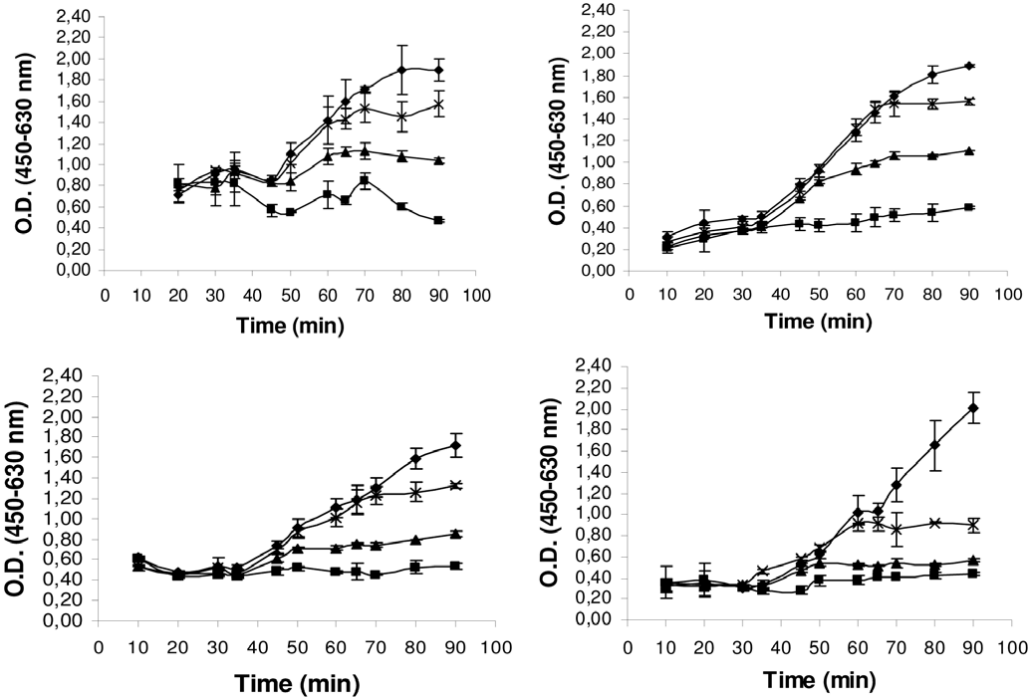
Effect of IxAC proteins on the deposition of C3b and factor B on agarose-coated plates. Loading human serum on agarose-coated microplate wells activates the alternative complement pathway. Purified recombinant IRAC II (A–B) or IxAC-B1 (C–D) were added after 30, 45 or 60 minutes. The reactions were stopped at various times. C3b and factor B deposition was detected using anti-C3 antibody (A–C) or anti-factor B antibody (B–D), respectively. Black diamond: no added protein; black square: 30 min.; black triangle: 45 min.; cross: 60 min.

We also tested the ability of IxACs to inhibit activation of the AP on agarose-coated ELISA plates using human serum as a source of complement factors. This experimental set-up is closer to physiological activation of complement than protein-protein interactions conducted on plastic surfaces. Recombinant IRAC II and IxAC-B1 were added in the assay at various time-points after addition of human serum (Figure 9).

The results indicated that IxAC proteins prevented C3b and factor B deposition in agarose-coated wells when added together with serum (not shown). They also stopped further C3 and factor B deposition when they were added at various times (30, 45 and 60 min) after initiation of the reaction as shown by the immediate plateauing of the curve. Nevertheless, they did not displace bound C3 or Bb as the measured amounts of bound factors did not drop (Figure 9). The results also showed that members of the two IxAC subfamilies are able to inhibit the formation of C3 convertase (Figure 9A–B and Figure 9C–D) in a similar manner. Although they were able to stop the ongoing formation of C3 convertase they could not undo previously formed complexes.

An additional experiment was also performed. After 60 min. of reaction, the reaction medium was replaced with fresh buffer containing the recombinant proteins but no human serum. A drop in the amount of bound Bb was observed in the presence of IRAC II or IxAC-B1 but not Iris (not shown).

These results therefore confirmed that IxAC proteins are able to inhibit the activation of the AP on a surface by preventing deposition of C3b and Bb.

### Class specificity is observed within Vertebrates

We next tested the hypothesis that diversification of anticomplement proteins helps *I. ricinus* counteract the complement activity of its diverse host organisms. Freshly prepared sera from different vertebrate species were first titrated in the AP assay in order to define the volume causing 50% hemolysis (AH50). A wide range of AH50 values were observed, from the equivalent of 0.25 µl per microwell test (50 µl final volume) for *Boa constrictor* to 7.0 µl per test for Balb/c mice (Table 5). Heat-inactivated samples completely lost their hemolytic activity, confirming that this activity was indeed due to complement (not shown).

**Table 5:** Host specificity of anticomplement activity by recombinant IxACs from *I. ricinus*.

Serum source	AH50	Irac I	Irac II	IxAC-B1	IxAC-B2	IxAC-B3	IxAC-B4	IxAC-B5	RaHBP2
1. *Homo sapiens* (4)	2.4	+	+	+	+	+	+	+	−
2. *Canis familiaris* (3)	1.7	+	+	+	+	+	+	+	−
3. *Ovis aries* (5)	2.3	+	+	+	+	+	+	+	−
4. *Sus domesticus* (2)	4.5	+	+	+	+	+	+	+	−
5. *Bos Taurus* (3)	1.7	+	+	+	+	+	+	+	−
6. *Cervus elaphus* (3)	0.8	+	+	+	+	+	+	+	−
7. *Mus musculus* (10)	7.0	+	+	+	+	+	+	+	−
8. *Phasianus cochicus* (5)	1.3	−	+	−	−	−	−	−	−
9. *Gallus gallus* (4)	2.2	−	−	−	−	−	−	−	−
10. *Meleagris gallopavo* (1)	1.6	−	−	−	−	−	−	−	−
11. *Columba liva* (5)	1.0	−	−	−	−	−	−	−	−
12. *Tropidurus torquatus* (2)	1.50	−	−	−	−	−	−	−	−
13. *Boa constrictor* (1)	0.30	−	−	−	−	−	−	−	−
14. *Elaph guttata* (3)	0.20	−	−	−	−	−	+	−	−

We assessed the ability of individual recombinant IxACs proteins to inhibit the alternative pathway (AP) of complement in sera from various vertebrate species as indicated. AP activation was assessed by hemolysis of added rabbit erythrocytes. (+) and (−) indicate the existence or absence of a dose-response relationship between added recombinant proteins and percent inhibition of hemolysis. AH50 value, serum amount (µl) that causes 50 % hemolysis of rabbit erythrocytes in 50 µl of the AP assay. Host N-° 1–7: mammals; 8–11: birds; 12–14: Squamates. Values between brackets indicates the n° individuals from which the serum pools were constituted.

Identical amounts of normalized IxAC proteins from *I. ricinus* and control RaHBP2 were then added to the AP hemolysis assay in the presence of AH50 volumes of serum. The 7 anticomplement proteins reproducibly inhibited all mammalian sera in a dose-dependent manner (Table 5 and Figure S3). In some species, such as humans (Figure 3) or *B. Taurus* and *M. musculus* (Figure S3) the dose-response curves were similar for the seven IxACs from *I. ricinus*. In other mammals such as, *C. familiaris*, *O. aries*, *S. domesticus* and *C. elaphus* (Figure S3) lower doses of the proteins had different efficiencies. They all reached similar plateau values at higher doses of protein, though. We also observed that most species were not equally sensitive to IxAC inhibition of the AP. Thus hemolysis of rabbit red blood cells by mouse serum was inhibited by not more than ∼30% whereas the hemolytic activity of human serum was inhibited at ∼85 %. Intermediate plateau values were observed for the other species tested (Figure S3). On the contrary, IxACs from *I. ricinus* did not affect most bird and squamate reptile sera, with the exception of IRAC II and IxAC-B4, which inhibited AP activity of one bird (*Phasianus colchicus*) and one snake (*Elaph guttata*), respectively.

To summarize, all 7 IxAC from *I. ricinus* inhibited the AP in all mammal species tested. Inhibition of the AP in only one bird by only IRAC II and only one squamate species by only IxAC-B4 was also observed.

### Expression patterns of individual *I. ricinus* IxACs

Primer pairs designed to specifically detect each IxAC messenger (Table S3) were used to analyse their pattern of expression by RT/PCR in individual ticks, at various stages of their life cycle and during the bloodmeal.

**Figure 10:**
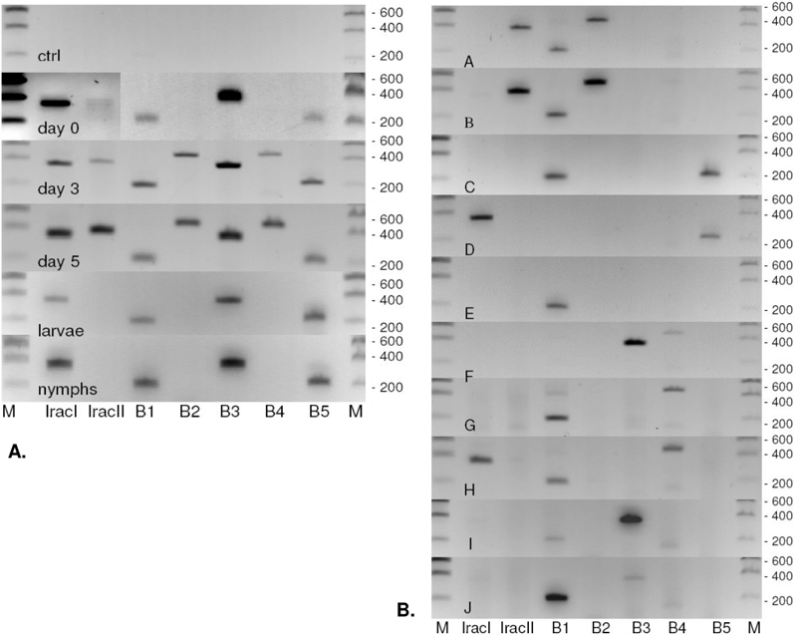
Expression patterns of individual IxACs. PolyA+ RNA was extracted from various *I. ricinus* material as indicated and reverse transcribed. The resulting cDNAs were submitted to PCR analysis using pairs of primers specific for the indicated IxACs. Non- reverse transcribed polyA+ RNA from the pool was included as negative control (Ctrl). PCR products were run on 1.2 % agarose gels. M, DNA size markers. B1 to B5, IxAC-B1 to IxAC-B5. Sizes in bp are indicated. Panel A: Analysis of salivary glands of a tick population (70 specimens, pool) and from individual female ticks at day 5 of the bloodmeal (A to J). Panel B: Analysis of pooled salivary glands of tick female populations at day 0 (25 specimens), day 3 (25 specimens) and day 5 (70 specimens) of the bloodmeal as well as from pooled gorged nymphs (25 specimens) and larvae (25 specimens).

We first investigated IxAC expression during the life cycle. All 7 IxAC sequences were detected in polyA+ RNA from the original pool of 70 salivary glands from adult females. No PCR product was observed from poly A+ RNA that had not undergone reverse transcription, indicating that we did not amplify fragments of genomic DNA (Figure 10a). Younger stages of the life cycle (larvae and nymphs) expressed IRAC I, IxAC-B1, B3 and B5 (Figure 10b). We also investigated whether they expressed additional anticomplement proteins not found in adults by searching for the full coding sequences in 10 pooled nymph and 10 pooled larvae cDNA in high-fidelity conditions using primer pairs designed from the 5′ and 3′ ends of IxAC-A or IxAC-B coding sequences (Table 1). Primers amplified the expected ∼600 bp products which were then inserted into pCRII and sequenced. Only IRAC I was retrieved from nymphs (7 independent clones), and IxAC-B1 and IxAC-B5 from larvae (2 and 1 clones respectively). The “nymphal” or “larvae” nucleotide sequences were 100% identical to “adult” ones.

We then investigated the expression of the IxAC repertoire during the bloodmeal. Messengers for IRAC I, IRAC II, IxAC-B1, IxAC-B3 and IxAC-B5 were detected in unfed females and the complete repertoire was detected in salivary glands from females on day 3 of the bloodmeal and on day 5 (Figure 10b).

When analysing the pattern of IxAC expression in 10 individual adult females we found that no single specimen expressed the whole range of anticomplement IxAC proteins. Each individual tick expressed one (1 tick), two (6 ticks) or three proteins (3 ticks). IxAC-B1 was expressed in 8/10 ticks tested, followed by IxAC-B3 and B4 (3/10), then IRAC I, IRAC II, IxAC-B2 and B5 (2/10). Four individual ticks expressed members of both IxAC-A and IxAC-B and 6 individual ticks expressed only members of IxAC-B. None expressed solely members of IxAC-A.

Taken together, the results indicated that IxAC are expressed in all ticks tested, throughout the bloodmeal and at all the development stages examined. There was no evidence for stage-specific variants although some members of the family may be induced or up regulated during the first days of the bloodmeal and in the adult. Individual females expressed individual members of the family and no individual tick expressed the complete series of *I. ricinus* IxACs.

### Antigenic diversification in the IxAC family in *I. ricinus*

**Figure 11:**
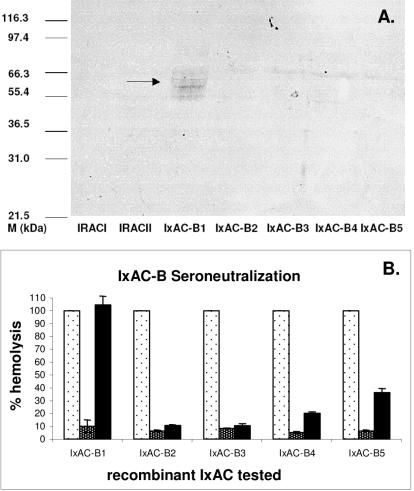
Antigenic specificity of recombinant IxACs from *I. ricinus*. Standardised amounts of the seven *I. ricinus* recombinant IxACs were analysed for antigenic specificity. Panel A. Western blot analysis. The serum from a mouse immunized against IxAC-B1 by genetic immunization followed by a protein boost recognised solely recombinant IxAC B1. M, molecular weight markers (Mark12, Invitrogen). Panel B. Seroneutralization experiments. AP hemolysis assays were conducted with and without the seven recombinant IxACs. 100% hemolysis was obtained in the absence of anticomplement protein (light dotted histogram). Recombinant IxACs alone (dark dotted histogram) or recombinant IxACs plus heat-inactivated sera from mice immunized against IxAC-B1 (anti IxAC-B1, black histogram) or mock immunized mice (anti-PBS, white histogram) were added as indicated. Neutralization of activity as indicated by a recovery of hemolysis, was observed only on IxAC-B1. Upper panels: seroneutralization of IxAC-A subfamily. Lower panel: seroneutralization of IxAC-B subfamily. Error bars represent standard deviations.

A monospecific mouse antiserum to IxAC-B1 was produced in mice by DNA immunization followed by a booster with purified recombinant IxAC-B1. It was used to perform western blot analysis of standardized amounts of IRAC I to IxAC-B5 (see Figure 3a) in parallel with the anti-V5 commercial antibody. As shown in Figure 11A, mouse anti-IxAC-B1 serum only recognized IxAC-B1 and none of the other IxACs from *I. ricinus*. This shows that epitopes recognized on IxAC-B1 in this assay were not present on any other member of the family.

The neutralizing potential of these antibodies was also assessed. Standardized amounts of 7 recombinant IxACs from *I. ricinus* were pre-incubated with heat-inactivated anti-IxAC-B1 serum before assessing their ability to interfere with complement activity. Neutralization of AP inhibition activity as indicated by recovery of RBC lysis was observed only against IxAC-B1 (Figure 11B). Seroneutralization of AP inhibition by IxAC-B1 was not observed with pre-immune sera or with antisera directed to the unrelated protein Iris (not shown).

These data show that an antiserum raised against one member of the IxAC family is able to recognize and functionally inhibit this member alone and no other member of the family. It suggests that immunodominant epitopes of a member of the family are not shared by other members.

## Discussion

The interaction between hard ticks and their hosts is a good example of an ongoing “arms race” between a parasite and its host. As the tick feeds on its vertebrate host for periods of several days it must counteract all the host's defense mechanisms including hemostasis, inflammation and the immune response. This is accomplished by injecting batteries of active proteins in the saliva. Most are coded by multigene families only a few of which have been fully characterized. In this work we have attempted to provide an exhaustive analysis of one such family, the family of salivary inhibitors of the alternative complement pathway in *Ixodes ricinus*. Our analysis includes: i) a complete or nearly complete inventory of members of the family, ii) a detailed study of its action mechanism: these proteins bind to properdin, a positive activator of complement, thereby preventing early rejection of the tick by the innate response, and iii) investigation of the possible selective pressure causing diversification: sequence diversification comes with antigenic diversity and helps circumvent the host's adaptive immune response.

### A large family of complement inhibitors in the hard tick *I. ricinus*

We describe five new anticomplement sequences (AM407396 to AM 407400) from the hard tick *Ixodes ricinus*. These share less than 40 % amino-acid identity with previously described ISAC of *I. scapularis* [25] and with IRAC I and IRAC II from *I. ricinus* [31]. The new sequences clustered in a distinct group of anticomplement sequences as indicated by phylogenetic analysis (Figure 1). We also proposed the acronym “IxAC” for *Ixodes* Anti-Complement to the larger family of *Ixodes* anticomplement proteins related to prototypical ISAC.

Our analysis provides a reasonably exhaustive and accurate inventory for the tick population under study. This is supported by several lines of evidence: i) 12 RT/PCR inventories performed independently on two different cDNA using 6 different primers pairs consistently yielded the same set of sequences; ii) Sequences identical to previously described IRAC I and IRAC II were recovered; iii) IxAC-B1 and IxAC-B2 were first discovered by serendipidity and then repeatedly found again by RT/PCR analysis of salivary gland cDNA. The existence of additional variants (either alleles or paralogs) in the larger *I. ricinus* species may be investigated on specimens captured in the field or on specimens from other laboratory colonies.

We also noticed consistent differences in the frequency of the 7 *I. ricinus* IxACs in RT/PCR inventories (Table S1). This may be due to the differential expression of IxAC genes in individual ticks (Figure 10). Alternatively, different steady-state levels of messengers may also be responsible.

The following arguments are consistent with the theory that the 7 *I. ricinus* IxAC genes are paralogs rather than alleles: i) The 7 *I. ricinus* IxAC proteins are not very closely related (65% amino-acid identity at most), ii) They could not be grouped together into a single phylogenetic cluster, iii) They were cloned from a restricted tick population, iv) Some individual female ticks expressed up to 3 IxACs simultaneously, indicating the existence of at least two loci.

Daix et al. [31] developed specific monoclonal antibodies that can differentiate IRAC I and IRAC II by immunofluorescence analysis on dissected salivary glands. They observed co-expression of the two proteins in the salivary glands of 12 adult specimens collected in various woodland locations throughout Belgium. The authors concluded that IRAC I and IRAC II are not alleles but rather co-expressed members of a multigene family. However, in our RT/PCR analysis, IRAC I and IRAC II mRNA were detected together only twice in 10 individual specimens from the Neuchâtel breeding colony. This discrepancy may be due to cross detection of other members of the IxAC families by monoclonal antibodies directed against IRAC I and IRAC II.

### Phylogenetic analysis

Phylogenetic analysis of the nucleotide and amino-acid sequences by distance and maximum likelihood methods (Figure 1) indicated that all known *Ixodes* anticomplement proteins (IxACs) could robustly be grouped into two large groups or subfamilies: IxAC-A and IxAC-B. IxAC-B only contains our five new sequences from *I. ricinus* whereas IxAC-A contains all the other sequences. IxAC-A can be further subdivided into “ISAC-like” and “Salp9-like” clusters after the name of the founder sequences. Regardless of the method used, placement of IRAC I within the ISAC-like or Salp9-like cluster was not supported by bootstrap analysis. It therefore probably represents a third cluster within the IxAC-A family.

The presence of IxAC sequences in *I. ricinus*, *I. scapularis* and *I. pacificus* indicated that their last common ancestor and possibly the ancestral *Ixodes* tick possessed an IxAC-like sequence. The lack of *I. scapularis* and *I. pacificus* representatives in IxAC-B is best explained by methodological differences when looking for IxAC sequences in these two species. Indeed, we performed a dedicated family-specific inventory in *I. ricinus* whereas the *I. scapularis* and one *I. pacificus* entries are the result of large-scale untargeted sequencing of large numbers of clones taken at random or PCR screening using unique primers designed from ISAC and Salp20. Dedicated RT/PCR inventories of IxAC sequences in *I. scapularis* and/or *I. pacificus* would help resolve this question by showing whether or not the two species express members of IxAC-B.

IxAC homologs were not detected by RT/PCR analysis of *R. appendiculatus* salivary gland cDNA or by *in silico* interrogations of collections of available non-*Ixodes* (Metastriata) sequences. IxAC homologs, if any, in *R. appendiculatus* might be too divergent to be amplified by our PCR primers. Besides, the present coverage of Metastriata genomes in public databases may be very limited. On the other hand, our finding is consistent with the absence of reported inhibition of the alternative complement pathway by Metastriata.

### Positive (diversifying) selection of coding sequences

Since Ohno [41], gene duplication has been considered to be an important factor in evolution as it leads to the evolution of new gene functions. In higher organisms most genes belong to families of related genes formed by repeated gene duplication events during evolution [42].Considerable research is currently being carried out in order to understand the forces leading to and shaping multigene families in living organisms [43].Ticks are particularly suited for this analysis as their salivary proteins are coded by multigene families, a feature probably related to their bloodfeeding lifestyle.

As far as salivary proteins in general are concerned, the process of gene duplication accompanied by positive selection leading to the acquisition of novel protein functions is documented in snake venom proteins [44], [45] and in soft tick salivary proteins [46]. In the bloodfeeding diptera *Lutzomyia longipalpis,* variants of the maxadilan protein retain the same function and biochemical properties [47] but have undergone diversifying selection (Lanzaro, personal communication). This is associated with antigenic diversity leading to escape from the host's antibody response [48], [49].

We compared the coding sequences of the seven *I. ricinus* anticomplement sequences with one another as well as with a putative reconstructed ancestral sequence. We observed that percent identities between IxAC amino-acid sequences were consistently lower than percent identity at the nucleotide level. In addition, dN/dS ratios were consistently higher than 1. We concluded that IxAC coding sequences had been subjected to strong positive selection within the *I. ricinus* species. In other words, diversification of the IxAC amino-acid sequences was strongly selected for and this was not associated with speciation. This confirms the conclusion of a restricted analysis conducted on only two members of the family: Daix et al. [31] observed an excess of non-synonymous changes when they compared IRAC I, IRAC II and anticomplement sequences from *I. scapularis* and *I. pacificus* (i.e. members of the IxAC-A subfamily).

We examined the type of the selection pressure operating on the IxAC family in *I. ricinus* by specifically investigating the following possibilities i) structural and biochemical diversity, ii) mechanism and functional diversity, iii) adaptation to host species, iv) stage specificity, v) antigenic diversity.

### Structural and biochemical diversity

We first assessed the differences in the biochemical properties of the members of the IxAC family. Predicted and experimental biochemical properties of the seven proteins were analysed and compared to those of ISAC from *I. scapularis* and ISAC-I from *I. pacificus*. We observed very similar values for calculated pI and observed and calculated molecular weights. All recombinant proteins were exported in the supernatant as predicted by specialized algorithms. They were highly glycosylated and disulphide bonds were predicted in all of them (this article and ref. [25], [29]). Finally, comparison of the distribution of hydrophobic clusters in the amino-acid sequences predicted identical or very similar folds or tertiary structure in all IxACs.

### Mechanism and function diversity

The kinetics of inhibition of the alternative complement pathway in human serum were similar for all 7 recombinant IxACs from *I. ricinus*. The classical pathway was not affected. This is also a feature of the *I. scapularis* anti-complement proteins ISAC [25] and Salp20 [29] and it has been reported recently for *I. ricinus* IRAC I and IRAC II by Daix et al. [31]. We then investigated the action mechanism of the 7 *I. ricinus* IxACs. Protein binding and competition experiments suggested that all 7 proteins had the same action mechanism. This is consistent with the similar properties of members of the family and also with the identical or very similar predicted folds or tertiary structures. We conclude that IxAC sequence diversification is not primarily driven by selection for different biochemical properties and tertiary structure, or a change in the roles of the proteins. Minute differences are possible but they remain to be investigated.

Our study of this action mechanism showed that IxAC molecules bind specifically to properdin, preventing its association with C3 and thereby reducing formation of C3 convertase complex to levels observed in the absence of properdin. They also induce destabilization of pre-formed C3bBbP convertase. They consequently inhibit the activation of complement via the alternative pathway. Properdin (factor P) is known to increase ten times the half-life of C3bBb convertase although functional convertase activity may be obtained in its absence [12], [50], [51]. The effect of IxAC proteins may therefore be explained by a decreased stability of C3 convertase due to blocking of properdin. This is reminiscent of the effect of monoclonal antibodies that bind to properdin and knock out of the properdin gene in mice, and both lead to inhibition of the AP [19].To our knowledge this is the first time that direct interaction with properdin is described as a mechanism of complement regulation.

Two recent reports further emphasize the importance of properdin in the AP: Spitzer et al. [52] and Kimura et al. [53] showed that properdin can also bind directly to microbes, initiating assembly of C3BbP convertase and complement activation. Whether properdin is a positive regulator or an initiator of the AP, it is a central element and a critical molecular target for inhibitors. Nevertheless, it remains to be seen whether IxACs can also interfere with the binding of properdin to the target surface.

Ixodid ticks are pool feeders, they dilacerate small blood vessels at the bite site, generating a small haemorrhage or pool of blood in which saliva is injected and from which they pump blood [3]. Local inhibition of complement activation in the pool of blood is therefore beneficial for at least two reasons. Firstly, inhibition of the production of pro-inflammatory peptides will help prevent the inflammation response at the bite site; secondly, inhibition of MAC insertion on the mouthparts and midgut epithelium will prevent the destruction of tick tissue by complement factors present in the blood meal.

In order to obtain that effect, ticks must secrete sufficient amounts of the inhibitor. A very rough calculation of the amount required can be made. This must take into account properdin concentration (∼5 µg/ml in normal human plasma) [54], [12], the amount of ingested blood (little information is available about this volume which is probably several hundred microliters [3]), the amount of secreted saliva (unknown but limited by the amount of ingested blood as blood water is recycled into saliva) and the concentration of IxAC in the saliva. If there is complete recycling of blood water into saliva and an equimolar properdin-IxAC interaction, the IxAC concentration required may be estimated to be approximatively 1 ng/µl. However, the anti-complement effect needs only be local (i.e. in the immediate proximity of the mouth parts and midgut epithelium), and this drastically reduces the amount of complement to be neutralized. Moreover, the IxAC might not mediate the only anti-complement mechanism in *Ixodes* saliva. Inhibition of the IxAC expression in ticks by RNAi may help reveal additional anticomplement molecules.

Finally our results suggest that one molecule of IxAC may interact with several molecules of properdin. Thus, in the experiment shown in figure 7c, complete inhibition of binding of 200 ng of properdin to C3b was observed with around 25 nm inhibitor, corresponding to an IxAC/properdin molecular ratio of 1/3 to ¼. This is consistent with the finding that properdin is present in the serum as dimer, trimer and tetramer [55] forms. One IxAC molecule may therefore interact with one properdin polymer. This hypothesis is open to experimentation and is currently being tested in our laboratory.

The mechanism by which salivary gland extract (SGE) from *I. ricinus* inhibits the AP has been explored by Lawrie et al. [39]. They observed inhibition of the cleavage of fB to Bb and C3a production when rabbit erythrocytes were used as activators and complete human serum as a source of complement. Little or no C3b was observed on erythrocytes in the presence of SGE. Reconstitution of the alternative C3 convertase (C3bBb) *in vitro* from purified C3b, fB and fD (but not properdin) was not affected by SGE. Moreover, SGE had no effect on the cleavage of ^125^I-C3 into ^125^I-C3b by preformed C3bBb or when it was added simultaneously with C3b, factor B and factor D. These findings are similar to our results and may therefore be fully explained by the action of IxAC proteins in the saliva.

Lawrie et al. [39] also noticed that SGE provoked the cleavage of a ∼5 kDa peptide at the C-terminal end of purified C3 α chains. The product can apparently still be cleaved by preformed convertase to yield C3a. The authors also suggested that the larger fragment may not be able to participate in convertase formation. However, we were unable to reproduce this finding (data not shown). As saliva is a complex mixture of proteins, proteolytic cleavage of C3, as well as other possible additional mechanisms, may also be involved in inhibition of the AP.

Saliva, recombinant ISAC [25] and Salp20 [29] from *I. scapularis* as well as IRAC I and IRAC II from *I. ricinus* [31], have been previously tested for their ability to inhibit AP activation in experiments using human serum as source of complement and agarose as activating surface. The authors observed inhibition of the deposition of both C3b and Bb and release of pre-bound Bb but not pre-bound C3b from the plates. Release of C3b is not observed because this protein is covalently linked to agarose. Finally, recombinant Salp20 also inhibits the production of C3a and deposition of C3b on the surface of red blood cells in the AP hemolytic assay [29]. We conclude that the novel inhibition mechanism described here is consistent with published data concerning *Ixodes* tick saliva, salivary gland extracts, or recombinant anticomplement proteins.

### Comparison of IxACs with other anti-complement inhibitors

Complement is a critical component of innate and adaptive immunity mainly acting through specific cell lysis, the release of potent pro-inflammatory peptides and opsonization of target cells. To prevent tissue damage by over-activation, complement activation is subject to tight regulation by many physiological negative regulators. These include surface and soluble proteins such as Decay-Accelerating Factor (DAF/CD55), Complement Receptor-1 (CR1/CD35), and factor H. They compete with factor B for binding to C3b and facilitate dissociation of the C3bBb complex. Together with the membrane protein MCP/CD46, they also act as cofactors for factor I (fI), a serine protease leading to C3b inactivation by proteolysis. Finally, other membrane proteins such as CR2/CD21, protectin or CD59 protect the host cell membrane from inappropriate complement activation and cell destruction [9].

Many pathogens also target components of the complement system, sometimes taking advantage of existing physiological regulation mechanisms. Some pathogenic bacteria such as *Borrelia*, *Neisseria*, *Streptococcus* and *Yersinia* express receptors that bind host-derived soluble complement regulatory proteins, in particular factor H, FHL-1, and C4b binding protein [56]. Certain pathogenic viruses express proteins homologous to vertebrate complement regulators such as vaccinia complement control protein or apparently unrelated functional analogs [57]. Protein NS1 of West Nile Virus recruits soluble fH [58]. Surface protein gC of Herpes Simplex Virus binds to C3b and inhibits its association with C5 and properdin [59].

Parasitic protozoa such as *Leishmania* spp. and *Trypanosoma cruzi* are also able to counteract complement activation. In the former, the major surface protein GP63 is a protease that can cleave C3b to inactive iC3b. The latter expresses a 160 kDa homolog of DAF which binds to C3b and C4b and prevents formation of the convertase [60].

In the soft tick *Ornithodoros moubata*, lipocalin OmCI binds to component C5 and prevents interaction with C5 convertase or blocks the C5a cleavage site. It prevents generation of C5a and MAC and thus suppresses complement hemolytic activity while preserving the immune clearance and opsonization functions [33].

Cobra venom factor (CVF) is a homolog of cobra C3. It is a functional analog of C3b as it binds to factor B which is cleaved by factor D to form the CVF:Bb complex. This is a stable convertase that continuously cleaves component C3 and C5, leading to depletion of serum complement components [61].

Finally, complement is also considered as a privileged target for new therapeutic agents. They comprise serine protease inhibitors (as many components of the complement cascade are serine proteases), soluble versions of physiological complement regulators, chemical complement inhibitors, complement receptor antagonists and therapeutic antibodies [62]. For instance, the anti-C5 humanized antibody or eculizumab which inhibits the generation of both C5b and anaphylatoxin C5a is currently tested in clinical studies against autoimmune inflammatory diseases such as rheumatoid arthritis, glomerulonephritis and lupus erythematosus and has recently been shown to be effective against nocturnal paroxysmal hemoglobinuria [63].

As compared to these inhibitors, IxACs have a completely new action mechanism as they bind and prevent the action of properdin, a factor that has not yet been found to be negatively regulated. The only noticeable structural feature of IxAC proteins is the presence of 4 conserved cysteine residues predicted to make two disulphide bridges. These are also found in repeated, conserved motifs of ∼60 residues known as short consensus repeats (SCR's) characteristic of regulators such as fH [64]. Otherwise, there was no sequence homology or structural resemblance as indicated by the HCA method (data not shown). Moreover, no experimental determination of the 3D structure has yet been published and database interrogations yielded no hits with known 3 D structures (data not shown). Therefore, IxAC-mediated inhibition of the AP appears to be a novel mechanism both in terms of the molecular interactions involved and the structure of the inhibitor.

What would be the advantages of such a mechanism if IxACs are considered to be putative drugs? First, IxACs target the only physiological positive regulator of complement activation. Secondly, it acts very early in the AP cascade and in any case upstream of most known inhibitors. In addition, as properdin has recently been described to also be an initiator of AP [52], [53], IxACs may have a double action on AP, both on initiation and stabilization. Furthermore, properdin strictly acts at membrane level, mostly by stabilizing preformed C3 convertases complexes. Therefore IxAC inhibitors have a localized, highly targeted action, unlike many putative anticomplement drugs which have a systemic action [65]. Finally, it acts specifically on the AP, while many drugs on trial target either the classical pathway or, like C3 inhibitors, both the classical and alternative pathways. Even anti-properdin antibodies also have a significant inhibitory action on the classical pathway of complement activation [19]. The specificity of IxACs for the AP has two major consequences. Insofar as clinical treatment is considered, a major concern for anticomplement drugs is that by inducing a complete blockade of the complement system, they increase susceptibility to infections and/or trigger autoimmune-like symptoms [66]. The detrimental effects of complement inhibition would be significantly lowered by specific inhibition of the sole AP, with no action on most of the CP effects. This application is theoretically feasible as the only critical adverse effect of AP inhibition is an increased susceptibility to neisserial meningitis [62].

One limitation we foresee is the case of diseases in which the respective involvement of AP and CP are unknown or intricate. The therapeutical benefits of AP inhibition will then be difficult to evaluate.

### Absence of stage specificity

We also investigated the developmental and bloodmeal stage specificity of expression. In databases, *I. scapularis* IxAC entries are reported from adults and nymphs but no robust “adult-only” or “nymphal-only” cluster appeared in our phylogenetic analysis. Moreover, we found one instance where the same nucleotide sequence was reported in both stages. In *I. ricinus*, we detected IxAC messengers from both IxAC-A and IxAC-B by RT/PCR in all stages of the life cycle and there was no evidence for stage-specific sequences. However, not all seven IxACs were detected in larvae and nymphs, as we observed expression of only IRAC I, IxAC-B1, B3 and B5 in fed larvae and fed nymphs.

We also investigated the expression of the IxAC repertoire during the bloodmeal. IRAC I, IRAC II, IxAC-B1, B3 and B5 messengers were detected in unfed females and the complete repertoire was detected in salivary glands from pooled females at days 3 and 5 of the bloodmeal.

These results suggest that variability in the IxAC family is not due to the development of proteins specific for a development stage or a phase of the bloodmeal. It is still possible, however, that some members are induced or upregulated in the first days of the bloodmeal or in the adult. Alternatively, the apparent absence of certain messengers in unfed adults and in younger stages may be due to the fact that individual ticks expressed only one or a few IxAC. This may be answered by analyzing larger pools of larvae, nymphs and unfed adult salivary glands.

Finally, variability in IxAC expression by individual adult females was observed: individual adult females each expressed different individual or series (up to three) of IxACs although some variants such IxAC-B1 were expressed more often than others.

### Adaptation to host species

A rationale for IxAC variability may be the necessity for the tick to be able to inhibit AP activity from a wide range of hosts. Indeed, *I. ricinus* is known to infest a very large range of mammals, birds and some reptiles [3], [67] and it is possible that *I. ricinus* can infest any terrestrial vertebrate it encounters [68], [69].

Lawrie et al [24] compared the anticomplement activity of adult salivary gland extract (SGE) from several *Ixodes* species against a series of birds and mammals. The authors attributed the observed differences in anti-complement activity to host specificity of different tick species.

Recently, Schroeder et al. [70] compared the anticomplement activity properties of recombinant IRAC I and IRAC II on fresh serum from human, dog, horse, sheep, rat, pheasant and pigeon. They observed inhibition of human, canine, sheep, horse, pig and pheasant serum by both IRAC I and IRAC II. Neither inhibited pigeon or rat serum.

We extended this analysis to the seven IxACs in *I. ricinus* and a larger and more diverse panel of host species. Dose-dependent inhibition of the AP by all recombinant IxACs was observed in sera from all mammals tested. For birds, only pheasant serum was inhibited, by IRAC II alone. In addition, AP activity in the serum of the snake *Elaph guttata* was inhibited by IxAC-B4. Our results with IRAC I and IRAC II were identical to those of Schroeder except for pheasant serum where we noted no inhibition with IRAC I. However, the inhibition was very low in their study [70]. Small experimental differences may explain the different conclusion, i.e. “low level” rather than “absence” of inhibition.

As every IxAC can inhibit the AP in all mammals tested, we conclude that diversification is not due to host specificity within the *Mammalia* class. Rather, it appears that IxAC evolved as a mammal-specific pathway for AP inhibition. The fact that inhibition of AP activity is only induced by some IxACs in only one bird and one reptile might seem surprising given the very wide specificity of *I. ricinus*. This is fully explained, though, by the absence of properdin in Birds and Squamates as reported by Nonaka & Kimura [71]. IRAC II and IxAC-B4 might accidentally bind to other components of the complement system such as C6, C7, C8 and C9 of the MAC, as they also possess repeats of the TSR (TSP1) motif characteristic of Properdin [14]. Therefore, *Ixodes* ticks might rely on other mechanisms to inhibit the AP or complement effector mechanisms when feeding on most birds and reptiles (e.g. proteolysis of the C-terminus of the C3 α chain described by Lawrie et al. [39]).

Alternatively, as birds and reptiles have not been studied as exhaustively as mammals, it cannot be ruled out that properdin is present in some species or lineages but not in others. Besides, bird-specific and/or lizard-specific IxAC variants may not yet have been found. All the ticks used in this study have been bred on mice and rabbits in the laboratory for over 20 years from founder specimens initially captured in the surroundings of Neuchâtel in Switzerland. Therefore, they probably have a reduced genetic diversity and have been selected for efficient blood feeding on mammals. Tick specimens collected on infested birds and lizards will help resolve these questions.

Although all recombinant IxACs inhibited the AP in all mammals tested, quantitative differences appeared in the ability of various IxACs to inhibit the AP in a given host and in the ability of a given IxAC to inhibit the AP in different hosts. Sequence differences between the IxACs might result in species specificity. Indeed although the general 3D structure of IxACs appears to have been conserved, small changes might modulate their interaction with properdin. Preliminary analysis of properdin amino-acid sequences from different eutherian mammals (*H. sapiens*, *M. musculus*, *C. familiaris*, *E. caballus*) indicated a 75 to 79% identity between the mature proteins (B. Couvreur, unpublished). Although properdin is a very highly conserved protein in eutherians, it is conceivable that small differences in amino acid sequences may lead to changes in affinity for the inhibitors. Furthermore, sequence comparison between individual thrombospondin domains suggested that TSR5 is more variable than the other TSR domains (B. Couvreur, unpublished). Because TSR5 is involved in the interaction of properdin with C3b [55] and as IxAC displaces this interaction, it is tempting to speculate that TSR5 variability could lead to different affinities for IxAC inhibitors, different degrees of inhibition of C3b binding and differences in the level of inhibition of the AP hemolysis assay.

Alternatively, several other factors might also explain the observed differences between mammalian sera. These include i) different properdin concentrations in the plasma from different species, ii) different concentrations of C3 and other complement components, iii) different relative roles of properdin (stabilizer of C3 convertase versus activator of the AP) in the various species tested.

Whatever the explanation, these differences were not further investigated as they have no impact on the general conclusion of this study.

### Antigenic diversification

One of the most frequent countermeasures adopted by parasites against the host's immune system is antigenic variation. It allows the parasite to escape the adaptive response but also to prevent immediate rejection in a new host by an immune memory reaction following a previous contact.

Tick anticomplement proteins are immunogenic to mammals and antibodies are present in the serum of repeatedly infested rabbits and guinea pigs [28]. Furthermore, we showed here that mice antibodies can neutralize anticomplement activity. siRNA experiments on nymphs have shown that silencing ISAC -like molecules in *I. scapularis* leads to a dramatic reduction in bloodmeal survival of feeding nymphs [27] indicating the importance of anticomplement proteins for the parasite. An immune response to anticomplement proteins may therefore be a major threat to completion of the bloodmeal or even tick survival.

Our finding that members of the IxAC family of proteins in *I. ricinus* do not share immunodominant epitopes and that individual ticks express different IxAC variants indicate that part of a tick population are probably able to escape an anti-IxAC immune response. Antigenic variability may therefore be considered to be a selective advantage at population level. This is a likely explanation of why IxAC evolved in a multigene family undergoing diversification.

### Possible clinical roles of IxACs

Many diseases are caused either by an insufficiency or over-functioning of complement. The former mostly leads to increased susceptibility to infectious agents [62]. The latter is associated with a large number of diseases including inflammatory disorders (e.g. asthma, sepsis); disorders related to severe tissue injuries (e.g. burn injury, myocardial infarction, ischemia/reperfusion), auto-immune diseases (e.g. lupus erythematosus,glomerulonephritis, rheumatoid arthritis, psoriasis, multiple sclerosis) and degenerative diseases (e.g. Alzheimer's disease) [62], [65], [66]. In some cases, the involvement of complement is only circumstantial as activated components have been localized at the sites of clinical disease. In other cases the involvement of complement was deduced by the analysis of inhibitors deficiencies in animal models. For example, the AP is known to be involved in disorders such as ischemia-reperfusion injury (I/R), systemic lupus erythematosus (SLE), asthma and rheumatoid arthritis (RA) [72]. In mouse models of diseases, the fB-/- genotype is associated with no or reduced alterations (e.g. reduced deposition of C3b in kidney I/R injury) [73] confirming the role of AP and C3 convertase.

IxAC molecules may have a positive clinical effect in conditions where inappropriate complement activation involves the binding of properdin and C3b. This will lead to a reduced production of pro-inflammatory molecules, reduced deposition of C3b and reduced cell lysis due to deposition of the MAC. Again, in most complement-related disorders, the relative contribution of the AP and CP is not known. More information about these diseases is needed before IxAC can be used as candidate therapy. Therefore on the one hand, more information about these diseases is needed before the beneficial effects of IxAC can be evaluated. On the other hand, however, AP-specific inhibitors could not be restricted to diseases initiated by AP activation as, in many cases, the alternative pathway loop is triggered as a secondary effect to amplify the activation, irrespective of the initial trigger (AP, CP or lectin pathway). A definite indication for a putative role of IxACs as anticomplement therapy must await tests in animal models of candidate diseases. This is the subject of ongoing experiments in the laboratory.

IxACs may also be used as tools to investigate the role of complement activation in certain diseases. As its causal role is only suspected and not proved in many of these disorders, a specific inhibitor of complement activation may be used in animal models to determine this role. This was recently achieved in knockout mice deficient in some complement components. More specifically and interestingly, IxAC may make it possible to distinguish the respective contributions of the AP and CP in pathophysiological mechanisms.

### Concluding remarks

To summarize, in this study we described five new anticomplement proteins from the hard tick *Ixodes ricinus* and we report the first specific in-depth analysis of a tick multigene family. Our data suggest that this inventory of anticomplement sequences is complete for the investigated population. This provided an opportunity to study possible gene diversification mechanisms related to the acquisition of a bloodfeeding lifestyle by hard ticks. Diversifying selection was shown to operate. Nevertheless, all proteins had very similar biochemical properties. They all inhibited formation of C3 convertase by specifically binding to properdin. Differences in host specificity or patterns of expression could not account for sequence diversity. However, sequence divergence was associated with antigenic differences. As individual ticks do not express the same range of anticomplement proteins, certain individuals in a population would be able to escape the host immune reaction.

This is the first time that inhibition of the alternative pathway *via* the specific binding of a positive regulator is described. This is an efficient way of blocking the innate immune system and preventing early rejection of the tick by the host. Recombinant IxACs may therefore be useful tools for the investigation of the role of properdin in physiological and pathophysiological mechanisms. Because activation of the alternative complement pathway is involved in major human diseases such as ischemia-reperfusion injury, systemic lupus erythematosus (SLE), asthma and rheumatoid arthritis (RA), AP inhibitors may prove to be therapeutically beneficial [72]. To date, only two complement inhibitors have reached the pre-clinical testing stage: recombinant soluble complement receptor 1 (sCR1) [74] and anti-C5 monoclonal antibodies [75]. However, they do not act specifically on AP [19]. Selective inhibition of the AP by properdin inhibitors such as IxACs may therefore be used to treat pathological effects of uncontrolled complement activation by this pathway, without compromising the classical and lectin pathways.

## Materials and Methods

### Tick material

Specimens of *Ixodes ricinus* were raised in the tick breeding facility at the *Institut de Zoologie*, *Université de Neuchâtel* (Switzerland). Founders of the colony were initially collected in woodlands near Neuchâtel and have been maintained on rabbits (adults and nymphs) and SWISS mice (larvae) for over 20 years. Specimens of *Rhipicephalus appendiculatus* strain Mugaga were a kind gift of Dr Maxime Madder (Animal Health department, Prince Leopold Institute of Tropical Medicine, Antwerp, Belgium). The colony originated from individuals collected in East Africa. It was routinely maintained on rabbits. All specimens were devoid of transmissible pathogens.

Pairs of salivary glands from *I. ricinus* were dissected from i) 70 adult female specimens at day 5 of the bloodmeal; ii) 25 females at day 3 of the bloodmeal, iii) 25 unfed females, iv) 10 individual females at day 5 of the bloodmeal. Pools of 25 fully gorged larvae and 25 fully gorged nymphs were also prepared. In addition, salivary glands were prepared from 25 adult female and 25 adult male *R. appendiculatus*.

### Nucleic acid extraction and analysis

PolyA+ RNA was extracted from tick material using the Micro-Fasttrack 2.0 (Invitrogen).

All polyA+ RNA were reverse transcribed with Superscript III (Invitrogen) in the presence of RNaseOUT ribonuclease inhibitor (Invitrogen) using Not1-d(T)18 bifunctional primer (Amersham Biosciences). PolyA+ RNA from pooled day 5 salivary glands was also reverse transcribed with Superscript II (Invitrogen) using the GeneRacer oligodT reverse transcription primer (Invitrogen).

5′ and 3′ RACE experiments were performed using the Generacer kit (Invitrogen).

For restriction analysis, sequencing and small-scale transfection experiments, recombinant plasmid DNA was extracted using Genelute Plasmid Miniprep Kit (Sigma).

Plasmid constructs were sequenced using universal sequencing primers at Biovallée A.S.B.L. (Gosselies, Belgium) and at Genome Express (Meylan, France).

All commercial reagents and kits were used according to the manufacturers' instructions.

### RT/PCR inventories of IxAC sequences in *I. ricinus*

PolyA+ RNA extracted from 70 pooled pairs of salivary glands from *I. ricinus* females on day 5 of the bloodmeal was reverse transcribed using two different sets of reverse transcriptase and oligodT primers. cDNA1 was generated with Superscript II using the GeneRacer oligodT reverse transcription primer (Invitrogen). cDNA2 was produced with Superscript III (Invitrogen) in the presence of RNaseOUT ribonuclease inhibitor (Invitrogen) using Not1-d(T)18 bifunctional primer (Amersham Biosciences). Both were used for RT/PCR experiments designed to make an inventory of the family of anticomplement proteins in *I. ricinus*.

Upstream primers were designed manually from the 5′ end of coding sequences of anticomplement proteins available early in this project from *I. ricinus* (IRAC I and II, AM407396, AM407397: *IRI* primer) or from all available *Ixodes* spp. sequences (IRAC I and II, AM407396, AM407397, ISAC, Isac-1: *IXO* primer). Commercial downstream primers *Generacer 3′* (Invitrogen) and *Not1* (Amersham Biosciences) are designed to anneal to the 5′ end of the modified oligodT primers used for reverse transcription. In addition, downstream primers *UTR1* and *UTR2* were designed from the 3′ UTR of anticomplement sequences. All primers are listed in Table 1. They were purchased from Eurogentec (Liège, Belgium) and diluted to 10 µM in MillliQ water.

Six primer pairs were thus constituted: i) *IRI*-*generacer3′*, ii) *IXO*-*generacer3′*, iii) *IRI*-*Not1*, iv) *IXO*-*Not1*, v) *IXO*-*UTR1*, vi) *IXO*-*UTR2*. Primer pairs (i) and ii) were used on cDNA1 whereas primer pairs (iii) to (vi) were used on cDNA2 (Table S1). PCR amplification experiments were performed with the Expand High Fidelity Plus PCR System (Roche) in a final volume of 50 µl of commercial buffer containing 1.5 mM MgCl_2_. PCR cycling parameters were as follows: 2 min denaturation at 94°C, then 30 cycles of 30 sec at 94°C, 30 sec annealing at 61 to 65°C (depending on the Tm of the primer pairs), 1 min at 72°C, then final incubation for 7 min at 72°C in a PTC-100 Programmable Thermal Controller (MJ Research). Annealing temperature was set at the lower Tm for a given primer pair minus 5°C.

PCR products were routinely purified on polyacrylamide gels and inserted into both the pCRII and the pCDNA3.1/V5-His vectors (Invitrogen) by the TA method. Top10F' chemically competent *E. coli* cells (Invitrogen) were then transformed and plated onto LB-Agar-Ampicillin solid medium. Plasmid DNA from recombinant clones was analysed by *Eco*RI (pCRII) or *Bam*H1–*Xba*1 (pcDNA3.1/V5-His-TOPO) restriction and inserts ≥600 bp were sequenced on both strands.

Therefore a total of 2 different reverse transcription experiments, 6 different PCR amplifications and 12 ligations were performed during the course of RT/PCR inventories of anticomplement sequences in salivary glands of *I. ricinus* females.

### RT-PCR analysis of expression of individual IxACs

Pairs of PCR primers (Table S3) were designed from an alignment of the *I. ricinus* IxAC coding sequences to specifically amplify each of the family members one at a time, each primer pair generating a product of different size. They were synthesized by Sigma-Genosys and purified by HPLC. The specific messengers were searched by RT/PCR in polyA+ prepared from i) salivary glands of individual adult females at day 5 of the bloodmeal, ii) pooled salivary glands at day 0 of the bloodmeal (unfed females), iii) pooled salivary glands of females at day 3 of the bloodmeal, iv) pooled nymphs and v) pooled larvae.

PCR was performed using Taq polymerase in a 50 µl reaction volume according to the manufacturer's instructions (Roche Biochemicals). Except for IxAC-B4, PCR cycling conditions were as follows: 2 min denaturation at 94°C then 40 cycles of 30 sec at 94°C, 30 sec annealing at 57°C, 30 sec elongation at 72°C followed by a final 7 min. elongation at 72°C on PTC-100. This set of PCR conditions had to be slightly modified to amplify the specific IxAC-B4 fragment: 45 cycles and annealing at 60°C. PolyA+ submitted to reverse transcription without the actual RT enzyme were used as negative control template.

In parallel, family-specific reverse primers were designed from UTR or coding sequences to amplify the full coding sequences of members of IxAC-A (*IXO*-*CDSREV1*) and IxAC-B (*IXO*-*CDSREV2*) (Table 1). They were applied to cDNA from pooled nymphs and pooled larvae using the Expand-HF+ system for PCR amplification as described above.

### Expression and quantification of recombinant proteins

The coding sequences for *I. ricinus* IxACS were amplified by PCR and inserted into the vector pCDNA3.1/V5-His-TOPO (Invitrogen). Upstream primers were designed from the first 20 nt of each coding sequences. Nucleotides surrounding the ATG were changed to ACCATGG (IRAC I, IxAC-B1 to B5) or GCCATG (IRAC II) according to Kozak's consensus for efficient initiation of translation [76]. Downstream primers were designed from the 3′ end of the coding sequences omitting the TGA stop codon so as to create IxAC-V5His chimeras.

The coding sequence for *Rhipicephalus appendiculatus* histamine-binding protein 2 (RaHBP2) was also amplified from salivary gland cDNA of adult *R. appendiculatus* females using PCR primers designed from the original published sequence (U96081) [77] and inserted into vector pCDNA3.1V5His. Throughout this study, recombinant RaHBP2 was used as a negative control.

Subconfluent 293T cells in 35 mm diameter wells (Orange Scientific) were transfected with 2 µg plasmid DNA and 6.0 µl Fugene 6 (Roche Biochemicals) in Dulbecco's modified Eagle's medium (DMEM, Invitrogen) without FCS. The medium was harvested after 72h. Pooled supernatants were cleared by centrifugation, concentrated ∼50-fold by filtration on 30 kDa cut-off membranes (Millipore), dialyzed against GVB or VB buffers, and finally stored at –80°C in 40 µl aliquots.

Concentrated culture supernatants were analyzed by western blotting on a Hybond ECL membrane (GE healthcare) using an anti-V5 primary antibody (Invitrogen), an IgHRP conjugate as secondary antibody and the ECL detection reagent (GE healthcare) following the manufacturer's instructions. Autoradiogram signals were quantified with ImageQuant TL Software (GE Healthcare). The relative amounts of protein were adjusted by diluting the most concentrated IxAC protein to the level of the less concentrated one. After normalization, new western blot analyses showed not more than 2.5-fold differences in protein concentrations.

Samples were also subjected to N-deglycosylation using N-glycosidase F (New England Biolabs) using conditions recommended by the manufacturer.

The coding regions of IRAC II and IxAC-B1 were amplified by PCR (94°C for 30 s, 56°C for 30 s, 72°C for 1 min.; 30 cycles) using ExTaq DNA Polymerase (Taqara). The PCR product was inserted into the pBlueBac4.5/V5-His Topo vector (Invitrogen) in frame with the coding sequence of the V5 and His epitopes at the C-terminus. Recombinant baculoviruses were generated by recombination between pBlueBac/IxAC and Bac-N-Blue linear DNA virus (Invitrogen). Recombinant viruses were selected and amplified according to the manufacturer's instruction. Sf9 cells were infected with a high-titer stock of recombinant baculovirus and were incubated for 72 hours at 27°C in Sf900 II serum-free medium (Invitrogen). Recombinant IxACs proteins were purified from the cell culture supernatant by affinity chromatography on a His-Trap column (GE Healthcare). The proteins were recovered in 50 mM NaH2PO4 buffer (pH 7.5) containing 300 mM NaCl and 50 mM of imidazole. In experiments with purified proteins, we used protein Iris, a serpin from the salivary gland of *I. ricinus* [78], as a negative control because it was also expressed in the baculovirus/Sf9 and purified in the same manner.

### Computer-assisted analysis of sequences and database interrogations

Probable cellular targeting and eukaryotic leader peptide cleavage site were identified in amino-acid sequences with the Target P and Signal P programs respectively [79]. N-linked glycosylation and O-GalNAc (mucin type) glycosylation sites were predicted with the NetNGlyc 1.0 and NetOGlyc 3.1 [80] programs respectively. An hydrophobic anchor at the C-terminal end of the peptide sequence was searched for with the TMHMM v. 2.0 [81] program that predicts transmembrane helices in proteins. All were used online at the Center for Biological Analysis of the Technical University of Denmark (CBS prediction servers: http://www.cbs.dtu.dk/services/). Calculation of molecular weight and isoelectric point were performed with the Pepstats program from EMBOSS online at the European Bioinformatics Institute (http://www.ebi.ac.uk/emboss/pepinfo/). Putative S-S links were searched with program Disulfind [82] at http://cassandra.dsi.unifi.it/disulfind/.

Tick anticomplement amino-acid sequences were submitted to Hydrophobic Cluster Analysis (HCA) [83], [84]. Briefly, amino-acid sequences in an alignment are compared for the overall distribution of hydrophobic clusters, their sizes, shapes and orientations. They can also be compared for possible particular structures induced by specific residues.

The non-redundant, EST, GSS and PDB databases were interrogated online at the NCBI server (http://www.ncbi.nlm.nih.gov/BLAST/) using Blast family programs. Nucleotide and amino acid sequences of ISAC, IRAC I, IxAC-B1 were used as queries. We also interrogated the preliminary releases of the genome projects for non-*Ixodes* hard ticks *Amblyomma variegatum*, *Boophilus microplus*, *Rhipicephalus appendiculatus* online at the Institute for Genomic Research (http://tigrblast.tigr.org/tgi) and *A. americanum* available at the University of Oklahoma cDNA Blast Server (http://www.genome.ou.edu/tick.html).

### Phylogenetic analysis

Coding sequences were aligned using ClustalW under the Mega3 package to obtain inframe alignment of codons. Minor manual adjustments were made at the 3′ end (C-terminus) of the alignment. The Genedoc package [85](http://www.psc.edu/biomed/genedoc/) was used to visualize alignments and calculate percent identity of nucleotide and peptide sequences. Percent similarity of peptide sequences was also calculated with Genedoc using a Blosum 62 matrix. For phylogenetic analysis by the distance methods, we used programs in the Phylip 3.65 package (http://evolution.gs.washington.edu/phylip.html). The substitution model was F84 for nucleotide sequences and the JTT matrix for amino-acid sequences distance analysis. The PHYML 2.4.4 package [86] (http://atgc.lirmm.fr/phyml/) was used for maximum likelihood analysis. We used the HKY model for nucleotide sequence analysis and the JTT model for amino-acid sequences analysis. Transition to transversion ratios and proportions of invariable sites were estimated from the dataset. No γ distribution of rates among sites was applied. Bootstrap analysis was performed on 1000 replicates of the initial datasets. Trees were visualized with Treeview 1.6.6 [87]. Phylogenetic analyses were repeated on amino-acid and nucleotide alignments with or without the leader peptide sequences.

We used the Ancescon package to reconstruct a probable ancestral coding sequence [88] (http://protevo.eb.tuebingen.mpg.de/toolkit/index.phpviewancescon) from an alignment of the coding sequences for *I. ricinus* IxAC mature proteins. The dN and dS values were calculated using the Nei-Gojobori method as implemented in the MEGA 3.1 package [89] (http://www.megasoftware.net/).

### Serum samples

Complement assays were conducted with freshly prepared sera from the mammals, birds and squamates (snakes and lizards) listed in Table 5. All animals were healthy and non-immunized. Except for red deer, they had been maintained in confined environments and there was no evidence or history of tick bites.

Fresh blood samples were left to clot at room temperature for 2 to 6 hours. The sera were separated from the clot by centrifugation. They were then aliquoted and stored at –80°C. Hemolysed samples were discarded.

Human sera were obtained from four healthy male volunteers, Beagle dog (*Canis familiaris*) sera from three individuals, sheep (*Ovis aries*) sera from five individuals, pig (*Sus domesticus*) sera from two individuals, calf (*Bos taurus*) sera from three individuals, deer (*Cervus elaphus scoticus)* sera from three individuals. Ten female Balb/c mice (*Mus musculus*) purchased from Harlan Netherlands were bled by retro-orbital puncture. Chicken (*Gallus gallus*) sera were obtained and pooled from four individuals, pheasant (*Phasianus colchicus)* sera from five birds, domestic turkey (*Meleagris gallopavo*) serum from one individual, pigeon (*Columba liva*) sera from five individuals, lizard (*Tropidurus torquatus)* sera from two individuals, snake sera from one individual (*Boa constrictor*) and three individuals *(Elaph guttata).*

### Assay of the alternative complement pathway (AP)

Recombinant proteins were assessed for their capacity to inhibit the alternative complement pathway (AP) according to Giclas [90] on red blood cells (RBC) from naïve healthy female New Zealand White rabbits. Briefly, fresh sera were diluted in gelatin-veronal-EGTA buffer (GVB) in microwell plates and washed RBCs were added. The surface of rabbit erythrocytes activated the alternative complement pathway leading to their lysis and release of hemoglobin in the buffer. After 60 min incubation at 37°C, supernatants were recovered to measure absorbance at 415 nm with a Model 680 microplate reader (Biorad). The volume of serum causing 50% hemolysis (AH50 value) was then determined by serial dilutions and used for further tests.

1 to 10 µl of serum were introduced in the test depending of the host species considered. 100% lysis control consisted in total hemolysis produced by incubating 25 µl of MilliQ water. Background level (no hemolysis) was determined by incubating the erythrocytes in GVB buffer alone (without added serum). Each experimental point was done in triplicate and experiments were performed at least twice, by different investigators.

In order to test the inhibitory effect of the new proteins, up to 10 µl of standardized supernatant (see above) were introduced in the AP test. The inhibitor was serially diluted in a final volume of 25 µl GVB in the presence of AH 50 volumes of the host serum under consideration. The assay was then performed as described above. Percent inhibition of hemolysis was then calculated with the equation:
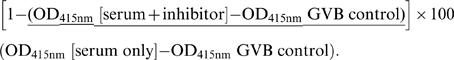



The kinetics of hemolysis inhibition were also investigated. The hemolytic AP assay was conducted as described above except that we added 200 ng of purified recombinant IRAC II, IxAC-B1 or unrelated protein IRIS to wells 10 minutes after the addition of RBC. Optical density at 415 nm was then taken every 5 or 10 minutes.

### Assay of the classical pathway (CP)

Recombinant proteins were also tested for inhibition of the classical complement pathway (CP) essentially as described by Colligan [91]. Ready-to-use reagents were purchased from Institut Virion\Serion GmbH (Würtzburg, Germany). They included sheep erythrocytes pre-coated with rabbit anti-sheep RBC antibodies and Veronal Buffer pH 7.3 (VB) containing NaCl, CaCl_2 _and MgCl_2_. Briefly, diluted serum was incubated in the presence of antibody-coated sheep RBCs in microplates. Immune complexes on the surface of RBCs activate the classical pathway of complement leading to lysis and release of hemoglobin. As in the AP assay, absorbance at 415 nm in the supernatant is proportional to the amount of lysed RBC. Pooled human serum was first titrated to determine the volume that produces 50 % hemolysis (CH50 value). Starting with 10 µl, standardized amounts of recombinant proteins were diluted two-fold in VB buffer containing the equivalent of 0.8 µl human serum per test (total volume 25 µl). Pre-coated sheep erythrocytes were then added and the reaction performed as described above. Results were expressed as percent inhibition of hemolysis as for the AP pathway.

### Western blot analysis of fB cleavage and C3a formation in the AP assay

The generation of peptide C3a from C3 and the cleavage of factor B in human serum during the AP assay in the presence of *I. ricinus* IxACs were assessed by western blot analysis as described by Lawrie et al. [39]. 10 µl of supernatant from AP assays conducted in the presence of *I. ricinus* IxAC or control protein RaHBP2 were analyzed by denaturing SDS/PAGE (C3 cleavage) or by non-denaturating PAGE (fB cleavage). Purified factor B was also included as a control in the analysis. Briefly, 10% acrylamide SDS/PAGE in tris–tricine buffer were run according to standard methodology. 10% acrylamide non-denaturating gel electrophoresis was conducted as described except that SDS and β-mercaptoethanol were omitted in all buffers. The material was blotted onto nitrocellulose sheets according to standard methodology. Rabbit anti-human C3a serum (Calbiochem, dilution 1∶5000) or goat anti-human factor B serum (Quidel, dilution 1∶1000) were used as primary antibodies. Anti-rabbit IgG or Anti-goat IgG horseradish peroxidase conjugates (Promega, dilution 1∶5000) were used as secondary antibodies. Blots were developed using the chemiluminescence substrate, ECL+ kit from Amersham (Bucks, UK) according to the manufacturer's instructions. Membranes were then exposed to KODAK X-ray film.

### Enzyme-linked Immunosorbent assay for measuring the binding of recombinant IxAC to components of C3 convertase

The binding of the seven recombinant *I. ricinus* IxAC proteins and control protein RaHBP2 to components of human C3 convertase was assessed using an enzyme-linked immunosorbent assay (ELISA). In some experiments, we also used recombinant IRAC II, IxAC-B1 and control protein Iris purified from the Baculovirus/Sf9 expression system.

Wells in 96-well polystyrene microtiter plates (Nunc) were coated with 200 ng of purified C3, C3b, factor B, factor D or properdin (Calbiochem) in 100 µl pH 7.4 phosphate-buffered saline (PBS, Invitrogen) overnight at 4°C. Wells were washed three times for 5 min. with washing buffer (8.1 mM Na_2_HPO_4_; 1.8 mM NAH_2_PO_4_; 0.05% Tween 20; 25 mM NaCl; 10 mM MgCl_2_) and blocked with PBS-Tween-BSA buffer (1% BSA, 0.1 % Tween 20 in PBS pH 7.4) for 1h at 37°C. Increasing amounts of recombinant *I. ricinus* IxACs and control proteins in 50 µl sample buffer (8.1 mM Na_2_HPO_4_; 1.8 mM NAH2PO4; 4% BSA; 0.05% Tween 20; 75 mM NaCl; 10 mM MgCl_2_) were added to the wells. After incubating for 1 h at 37°C, wells were washed 3 times with washing buffer. 100 µl antibody buffer (8.1 mM Na_2_HPO_4_; 1.8 mM NAH_2_PO_4_; 4% BSA; 0.05% Tween 20; 25 mM NaCl; 10 mM MgCl_2_) containing a mouse anti-V5 antibody (Invitrogen, dilution 1∶5000) were added to the wells. Plates were incubated for 1h at 37°C then washed again three times. Horseradish peroxidase-conjugated anti-mouse IgG antibodies (Promega, diluted 1∶7500) in 100 µl antibody buffer were incubated for 1h at 37°C. After 3 washes, 50 µl of 3,3′,5,5′ –Tetramethylbenzidine (TMB, Sigma) substrate was added. The reaction was stopped by adding 50 µl H_2_SO_4_ 0.2 N. Optical density at 450 and 630 nm was measured with a Model 680 microplate reader (Biorad). In a first series of experiments we tested the binding of purified recombinant IRAC II and IxAC-B1 and unrelated protein IRIS to C3 convertase components. In the second series we tested standardized amounts of the 7 *I. ricinus* IxAC and control protein RaHBP2 as expressed in the supernatant of transfected 293T cells (see above) on purified properdin alone.

We also assessed the effect of recombinant IRAC II, IxAC-B1 and unrelated protein IRIS on the binding of Properdin to C3b. Wells were coated with 150 ng of C3b. 200 ng factor P and increasing concentrations of IRAC II, IxAC-B1 or unrelated protein IRIS in sample buffer were added simultaneously to the wells. The amount of bound properdin was estimated using a primary mouse monoclonal antibody to factor P (diluted 1∶2000; Quidel) and horseradish peroxidase-conjugated anti-mouse IgG antibodies (diluted 1∶7500; Promega) using the above-described ELISA method.

### Enzyme-linked immunosorbent assays for assessing the effect of IxACs on the formation and stability of C3 convertase

We assessed the effect of recombinant IxAC proteins on the formation and stability of C3 convertase *in vitro* using the above-described ELISA method. 96-well polystyrene microtiter plates were first coated with 150 ng of C3b per well. The effect of IxAC proteins on the formation of C3 convertase was assessed by adding 200 ng of factor B, 20 ng of factor D, 200 ng of factor P in sample buffer to the wells together with increasing concentrations of IRAC II, IxAC-B1 or Iris. Plates were incubated for one hour at 37°C before antibody detection of fB or P. To assess the effect of IxACs on the stability of the C3 convertase, C3b-coated wells were incubated for 1 h with 200 ng of factor B, 20 ng of factor D, 200 ng of factor P in sample buffer. The wells were washed three times before deposition of IRAC II, IxAC-B1 or IRIS proteins. The plates were further incubated for one hour at 37°C. Anti-factor P (dilution 1∶2000) and anti-factor B (dilution 1∶1000) were used as primary antibody to detect bound proteins as described above. Results are expressed as % bound fB or properdin using the following formula:
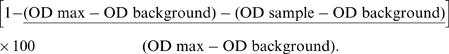



Background values were obtained by measuring OD values of C3-coated plates revealed with anti-fB or anti-properdin antibodies. OD max was measured by performing the test without added IxAC.

In a complementary experiment, we compared the effect of IRAC I, IxAC-B1 and the control protein Iris on the formation of C3 convertase in the presence (C3bBbP) or absence of properdin (C3Bb). 200ng of factor B, 20 ng of D, with or without 20 ng of properdin, and 200 ng of IRAC I, IxAC-B1 or control protein Iris were added to microtiter wells pre-coated with 150 ng purified C3b. The reaction was stopped at 0, 20, 40 and 60 min. and bound factor B or properdin were detected with the respective specific antibodies using the above-described ELISA method.

### Enzyme –linked immunosorbent assays for measuring C3 and factor B deposition on agarose-coated wells

The ability of *I. ricinus* IxACs to alter AP activation by a solid phase was assessed by agarose ELISA as described by Valenzuela et al. [25] with minor modifications. Deposition of C3b and fB on agarose-coated microplates was used to measure AP activation and C3 convertase formation. Briefly, wells of polystyrene microplates (Nunc) were coated with 100 µl of 0.1% melted agarose in water and incubated at 37°C for 48 h. Wells were then incubated with 50 µl of 200 mM HEPES, pH 7.4, 150 mM NaCl, 5 mM EGTA, 2 mM MgCl_2_, and 10% human serum for 90 min at 37°C. After 30, 45 or 60 minutes, 200 ng of purified IRAC II or IxAC-B1 was added to the plates. The reaction was stopped by washing five times with 200 µl of HEPES saline containing 2 mM MgCl_2_ and 10 mg/ml bovine serum albumin (HEPES-BSA buffer). Bound C3b and fB were revealed by Elisa. Goat anti-human C3 serum (Quidel, diluted 1∶5000) or goat anti-human fB serum (Quidel, diluted 1∶1000) used as primary antibodies were incubated for 1 h at 37°C in HEPES-BSA buffer. Wells were washed 5 times with 200 µl HEPES-BSA and IgG anti-goat peroxidase-conjugate (Promega, diluted 1∶5000) was then added as secondary antibody and incubated for 1 h at 37°C in HEPES-BSA. Plates were washed twice with HEPES-BSA, then twice with HEPES containing 10% Tween 20, and finally twice with HEPES buffer alone. 50 µl of 3,3′,5,5′ –Tetramethylbenzidine substrate (TMB, Sigma) was then added to each well and the developed color reactions were stopped by adding 50 µl 0.2 M H_2_SO_4. _Optical density at 450 and 630 nm was measured with a Model 680 microplate reader (Biorad).

### Immunization and antigen assay

IxAC-B1-specific antibodies were produced in Balb/c mice by DNA immunization followed by a booster protein injection. Briefly, 100 µg of plasmid pCDNA3.1/IxAC-B1_V5-His DNA in saline was injected four times in the anterior tibialis at three week intervals. Animals were then boosted once with 1 µg purified IxAC-B1 in Alum (Brenntag Biosector, DK). Sera were taken by retro-orbital puncture two to 5 weeks after the booster injection. Recombinant IxAC-B1_V5His protein was produced by transfecting subconfluent 293T cells with plasmid pCDNA3.1/IxAC-B1V5His in serum-free culture medium. The recombinant protein was purified from cell culture supernatants by chromatography on NiNTa columns (Invitrogen). Pre-immune and immune antisera were tested by western blot on the 7 *I. ricinus* IxACs and assessed *in vitro* for neutralization of anticomplement activity in the AP test. We reasoned that if seroneutralization occurred, antibodies would block inhibition of AP by IxACS and hemolysis would occur. 3.0 µl heat-inactivated mouse antisera were preincubated for 30 min at room temperature with 2.0 µl samples of standardized IxACS. The mixture was then added to 2.0 µl human serum in GVB buffer in a total volume of 25 µl. 25 µl RBC were then added and the AP assay was conducted as described above. Controls included i) sera from mice previously mock-immunized 3 times with PBS in Freund's adjuvant, ii) serum from mice immunized with the unrelated protein Iris [78], iii) GVB buffer. Results are expressed as % hemolysis as:
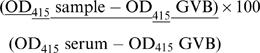



## Supporting Information

Figure S1 (0.14 MB TIF)

Figure S2 (0.36 MB TIF)

Figure S3 (0.37 MB TIF)

Table S1 (0.04 MB DOC)

Table S2 (0.07 MB DOC)

Table S3 (0.03 MB DOC)
