# Supplementary figures and images for: Variability and Action Mechanism of a Family of Anticomplement Proteins in Ixodes ricinus
Source: PLoS One. 2008 Jan 2;3(1):e1400. doi: 10.1371/journal.pone.0001400 (PMC2151134; doi:10.1371/journal.pone.0001400)

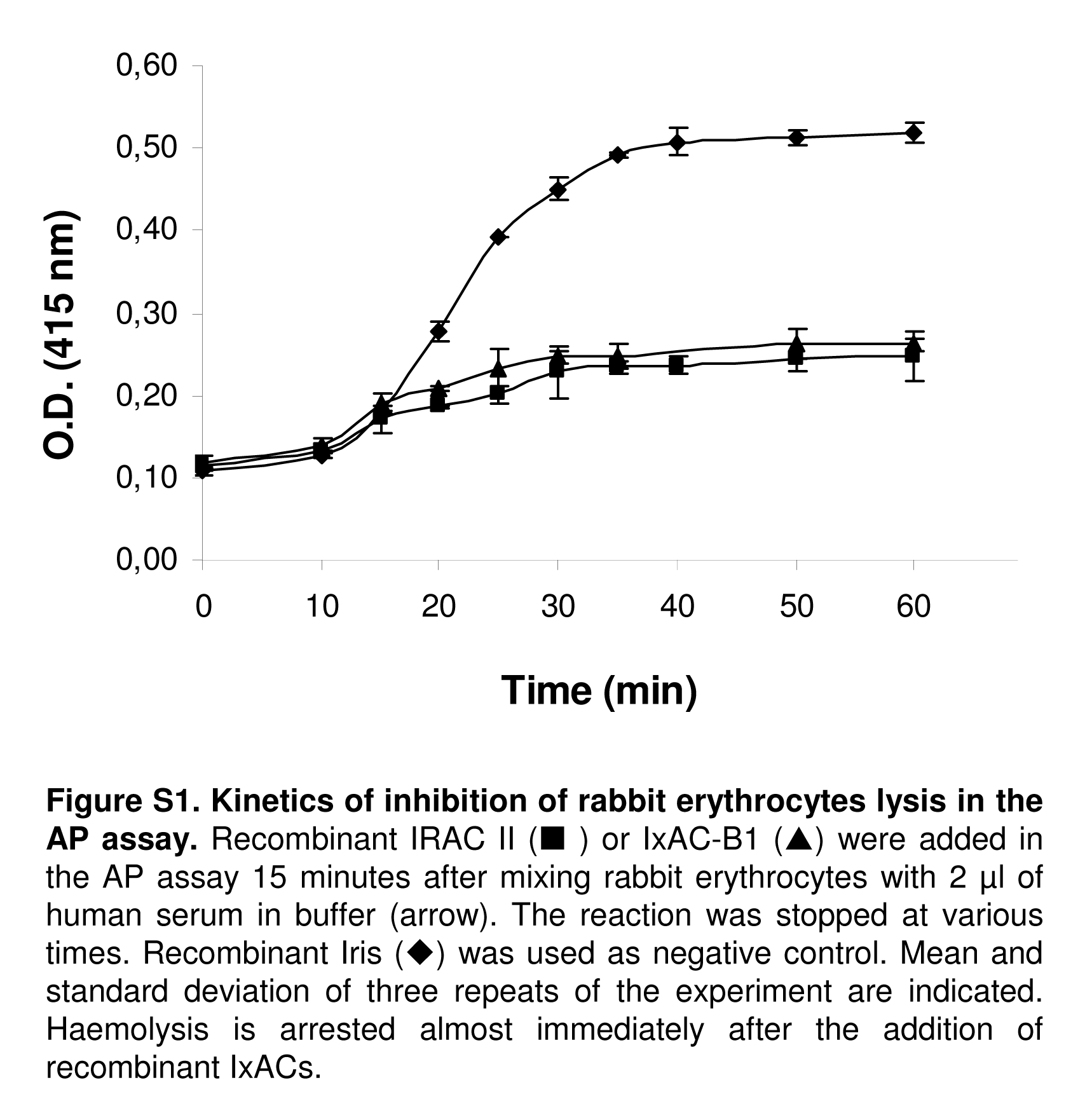

Supplement: Figure S1 — (0.14 MB TIF) [file pone.0001400.s001.tif]

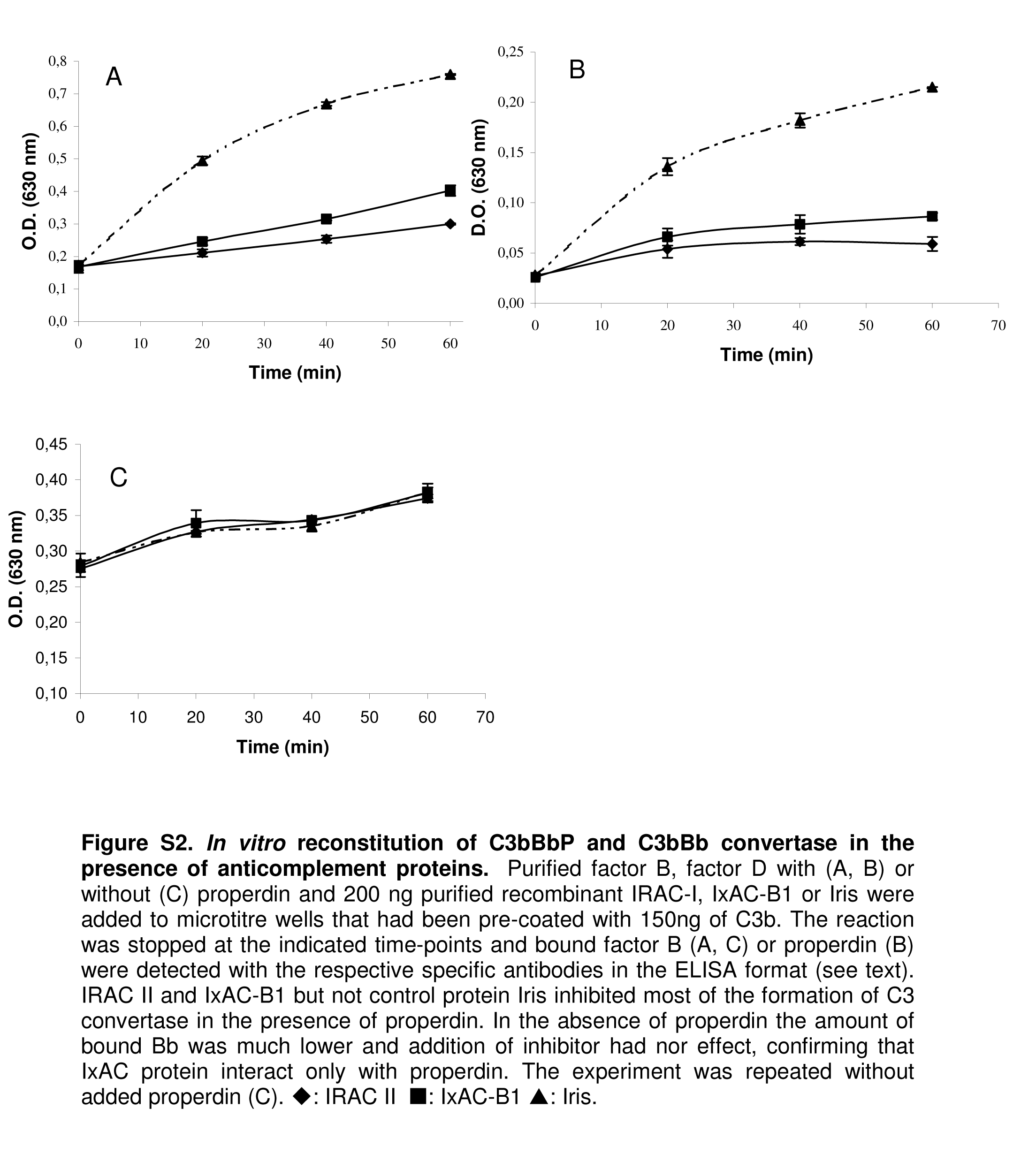

Supplement: Figure S2 — (0.36 MB TIF) [file pone.0001400.s002.tif]

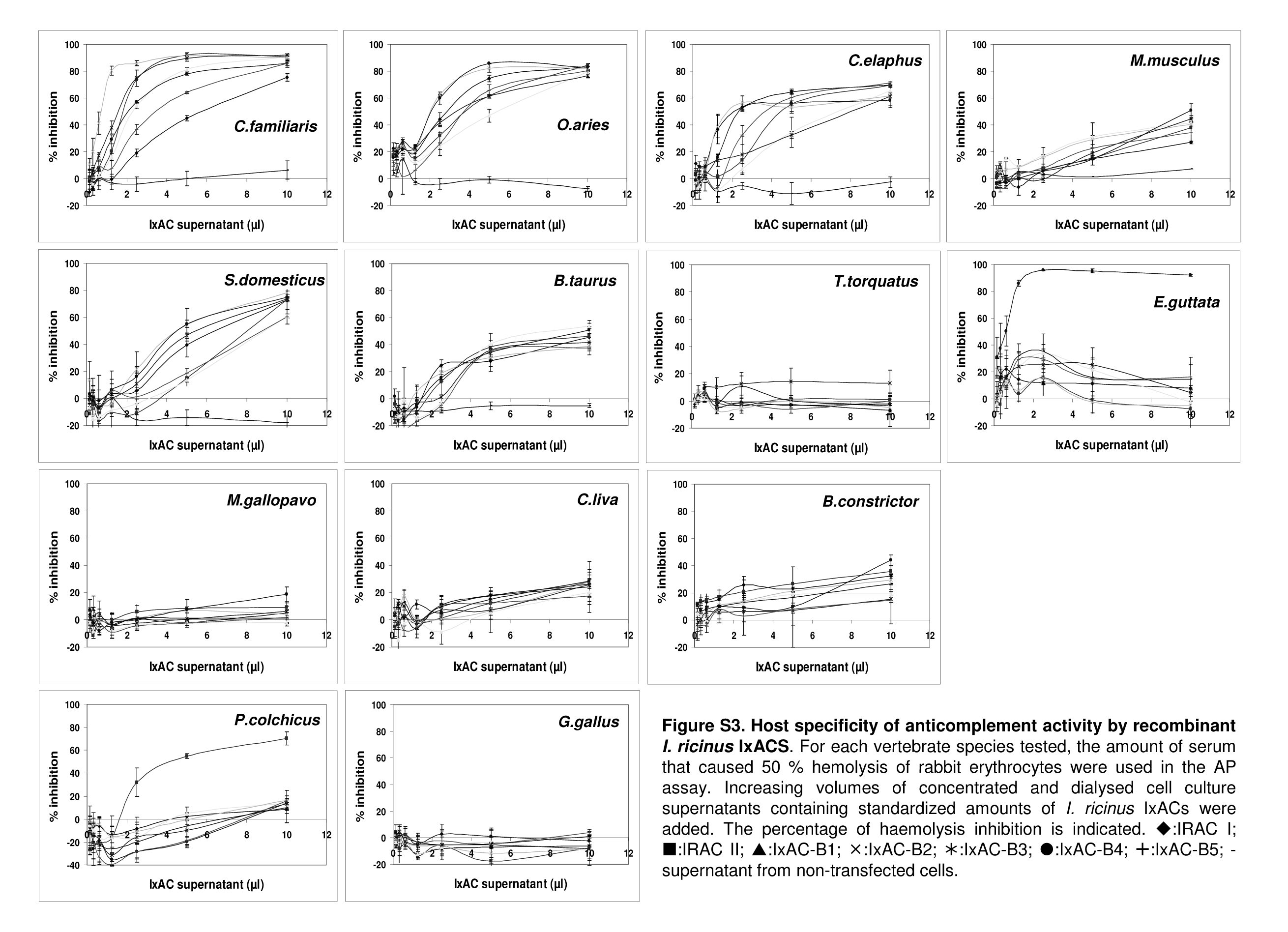

Supplement: Figure S3 — (0.37 MB TIF) [file pone.0001400.s003.tif]
