# Supplementary material for: Variability and Action Mechanism of a Family of Anticomplement Proteins in Ixodes ricinus
Source: PLoS One. 2008 Jan 2;3(1):e1400. doi: 10.1371/journal.pone.0001400 (PMC2151134; doi:10.1371/journal.pone.0001400)
Supplement: Table S1 — (0.04 MB DOC) [file pone.0001400.s004.doc]

**Table S1: Inventories of anticomplement sequences in *Ixodes ricinus* salivary glands.**

| Ligation N° | cDNA | PCR primer pairs | PCR | Cloned into | N | Number of sequences found | | | | | | | |
| --- | --- | --- | --- | --- | --- | --- | --- | --- | --- | --- | --- | --- | --- |
|  |  |  |  |  |  | I1 | I2 | B1 | B2 | B3 | B4 | B5 | NI |
| *1* | cDNA1 | IRI – 3’ generacer | PCR1 | pCDNA3.1V5His | 15 | 1 | 0 | 5 | 5 | 0 | 1 | 0 | 3 |
| *2* |  | idem | Idem | pCRII | 7 | 1 | 0 | 1 | 1 | 1 | 1 | 2 | 0 |
| *3* |  | IXO – 3’ generacer | PCR2 | pCDNA3.1V5His | 3 | 1 | 0 | 0 | 1 | 0 | 0 | 0 | 1 |
| *4* |  | idem | Idem | pCRII | 1 | 0 | 0 | 1 | 0 | 0 | 0 | 0 | 0 |
| *5, 7* | cDNA2 | IRI – Not1 primer | PCR3 | pCDNA3.1V5His | 23 | 2 | 0 | 15 | 2 | 2 | 2 | 0 | 0 |
| *6, 8* |  | Idem | Idem | pCRII | 16 | 3 | 0 | 9 | 2 | 0 | 2 | 0 | 0 |
| *9* |  | IXO – Not1 primer | PCR4 | pCDNA3.1V5His | 22 | 5 | 0 | 14 | 2 | 0 | 1 | 0 | 0 |
| *10* |  | Idem | Idem | pCRII | 18 | 4 | 0 | 6 | 7 | 0 | 1 | 0 | 0 |
| *11* |  | IXO – utr1 | PCR5 | pCDNA3.1V5His | 11 | 1 | 1 | 0 | 5 | 0 | 2 | 0 | 2 |
| *12* |  | IXO – utr2 | PCR6 | pCDNA3.1V5His | 6 | 0 | 0 | 3 | 2 | 1 | 0 | 0 | 0 |
|  |  |  |  |  | 122 | 18 | 1 | 54 | 27 | 4 | 10 | 2 | 6 |
|  |  |  |  |  | % |  |  |  |  |  |  |  | 4.9 |
|  |  |  |  |  | % | 15.5 | 0.9 | 46.6 | 23.3 | 3.4 | 8.6 | 1.7 | - |
| 12 ligations | 2 cDNAs | 6 primer pairs | 6 PCR experiments | 2 different vectors |  | 7 different IxAC genes | | | | | | | |

Two independent RT experiments were performed on polyA+ RNA from pooled salivary glands, generating two cDNA pools (cDNA1 and cDNA2). cDNA1 was produced with Superscript II using the GeneRacer oligodT reverse transcription primer (Invitrogen). cDNA2 was produced with Superscript III using the using Not1-d(T)18 bifunctional primer (Amersham Biosciences). A total of 122 clones from 6 separate RT/PCR experiments were sequenced in both orientations. N, number of clones sequenced; I1 and I2, IRAC I and II; B1 to B5, IxAC-B1 to IxAC-B5; NI, non-IxAC.
