# Supplementary material for: Variability and Action Mechanism of a Family of Anticomplement Proteins in Ixodes ricinus
Source: PLoS One. 2008 Jan 2;3(1):e1400. doi: 10.1371/journal.pone.0001400 (PMC2151134; doi:10.1371/journal.pone.0001400)
Supplement: Table S2 — (0.07 MB DOC) [file pone.0001400.s005.doc]

**Table S2: Database entries related to tick anticomplement protein ISAC.**

| A. From non-redundant database (28 entries recovered) | | | |  |
| --- | --- | --- | --- | --- |
|  |  |  |  |  |
| Accession | Tick Species | given name* | life cycle stage, organ | note on the sequence |
| AY878714 | I. ricinus | ***IRACI,*** | adult female, salivary gland, | complete cds |
| AY878715 | I. ricinus | ***IRACII,*** | adult female, salivary gland, | complete cds |
| AY674272 | I. pacificus | ***ISAC-1*** | adult female, salivary gland (d. 3-4) , | complete cds |
| AF209917 | I. scapularis | ***Salp20*** | engorged nymphs, salivary glands | complete cds |
| DQ066183 | I. scapularis |  | unspecified st., salivary gland, | complete cds |
| DQ065897 | I. scapularis |  | unspecified st., salivary gland, | complete cds |
| AF270496 | I. scapularis | ***ISAC*** | adult, salivary gland, | complete cds |
| AY956388 | I. scapularis | ***ISAC-like clone 122*** | nymphal, salivary gland (fed 72hrs on mice), | 5’ end of leader peptide missing |
| DQ066136 | I. scapularis |  | nymphal, probably SG, | complete cds |
| DQ066167 | I. scapularis |  | nymphal, probably SG, | complete cds |
| AY956387 | I. scapularis | ***ISAC-like clone 120*** | nymphal, salivary gland (fed 72 hrs on mice), | 5’ end of leader peptide missing |
| AY956386 | I. scapularis | ***ISAC-like clone 113*** | nymphal, alivary gland (72 hrs fed on mice), | large internal deletion |
| AF278574 | I. scapularis | ***Salp9*** | nymphal, salivary gland (engorged), | 3’ end of cds, half cds missing |
| EU008545 to EU008559 | I. scapularis | ***Salp20-like, protein 1 to 15*** | nymphal, whole or salivary gland (fed 48hrs) | complete cds |
|  |  |  |  |  |
| B. From EST_Others database (20 entries recovered) | | | |  |
|  |  |  |  |  |
| Accession | Tick Species | given name | life cycle stage, organ | note on the sequence |
| DN968378 | I. scapularis |  | adult female, salivary gland, | complete cds. |
| DN970152 | I. scapularis |  | nymphal, salivary gland, | complete cds. |
| DN974829 | I. scapularis |  | adult female, salivary gland, | complete cds. |
| DN970085 | I. scapularis |  | infected nymphal, salivary gland, | complete cds. |
| DN969177 | I. scapularis |  | infected nymphal, salivary gland, | complete cds, 1 ambiguous position. |
| DN968548 | I. scapularis |  | adult female, salivary gland, | complete cds. |
| DN970161 | I. scapularis |  | nymphal, salivary gland, | complete cds. |
| DN971112 | I. scapularis |  | nymphal, salivary gland, | start ATG missing. |
| DN970233 | I. scapularis |  | nymphal, salivary gland, | complete cds. |
| DN969639 | I. scapularis |  | infected nymphal salivary gland, | complete cds, 2 ambiguous positions. |
| DN969295 | I. scapularis |  | infected nymphal salivary gland, | complete cds. |
| DN971052 | I. scapularis |  | nymphal salivary gland, | first two codons missing at 5’ end. |
| DN967239 | I. scapularis |  | adult female salivary gland, | coding sequence incomplete at 3’ end. |
| DN970999 | I. scapularis |  | nymphal salivary gland, | coding sequence incomplete at 3’ end. |
| DN967646 | I. scapularis |  | adult female salivary gland, | coding sequence incomplete at 3’ end. |
| DN970431 | I. scapularis |  | nymphal salivary gland, | coding sequence incomplete at 5’ end. |
| DN967741 | I. scapularis |  | adult female salivary gland, | 3’ end missing (less than 50% of cds). |
| DN967649 | I. scapularis |  | adult female salivary gland, | 3’ end.missing |
| DN967728 | I. scapularis |  | adult female salivary gland, | 3’ end missing (less than 50% of cds). |
| DN970837 | I. scapularis |  | nymphal salivary gland, | 5’ end missing (only 150 nt at 3’ end). |

The non-redundant, EST, GSS and PDB databases were interrogated online at the NCBI server (http://www.ncbi.nlm.nih.gov/BLAST/) using members of the Blast family of programs. Nucleotide and amino-acid sequences of ISAC, IracI and Irac3 were used as queries. We also interrogated the preliminary releases of the genome projects of the non-*Ixodes* hard ticks *Amblyomma variegatum*, *Boophilus microplus*, *Rhipicephalus appendiculatus* online at the Institute for Genomic Research (TIGR, http://tigrblast.tigr.org/tgi) and *A. americanum* at the University of Oklahoma cDNA Blast Server (http://www.genome.ou.edu/tick.html). All queries yielded the same results. Additional data such as stages and organs of origin were recovered from the entry files or from the associated publications. (*) when available.
