# Supplementary material for: Variability and Action Mechanism of a Family of Anticomplement Proteins in Ixodes ricinus
Source: PLoS One. 2008 Jan 2;3(1):e1400. doi: 10.1371/journal.pone.0001400 (PMC2151134; doi:10.1371/journal.pone.0001400)
Supplement: Table S3 — (0.03 MB DOC) [file pone.0001400.s006.doc]

| Primer | Sequence | Tm | Targets | Amplicon size |
| --- | --- | --- | --- | --- |
| Ir1F | 5’ - Ggagagcaagatacaggagaga - 3’ | 66°c | Irac I | 329 |
| Ir1R | 5’ - GACACCTTTTCACTGTTTTCACC – 3’ | 66°c |  |  |
| Ir2F | 5’ - GGagagcgagagtcaggagt - 3’ | 64°c | Irac II | 376 |
| Ir2R | 5’ - GCTTCCTCTGCTTTTGTTATATC – 3’ | 64°c |  |  |
| B1F | 5’ - CTGgtgatttctatactatttttac – 3’ | 64°c | Ixac-B1 | 242 |
| B1Ra | 5’ - CTCACTGAGTGGCTTTTATAGG – 3’ | 64°c |  |  |
| B2F | 5’ - gttcttctaacgaacaagagtcaa – 3’ | 66°c | Ixac-B2 | 483 |
| B2R | 5’ - AGTGCTACTACAGCAGGCTTGA – 3’ | 66°c |  |  |
| B3F | 5’ - gaagaagttcaggaacaagagccag - 3’ | 64°c | IxAC-B3 | 347 |
| B3R | 5’ - GGAGTCTCCGTGGTAGCATC - 3’ | 64°c |  |  |
| B4F | 5’ - CTTctgaagaagaaacctaccata – 3’ | 66°c | IxAC-B4 | 481 |
| B4R | 5’ - AATGGCCTCTACAGCAGTTTCA – 3’ | 64°c |  |  |
| B5F | 5’ - CTGataatttcactctcatgtttaa - 3’ | 64°c | Ixac-B5 | 231 |
| B5Rb | 5’ - CCGCACACACCTCACGCAATT - 3’ | 66°c |  |  |

**Table S3: PCR primers for RT/PCR detection of individual IxACs from *I. ricinus*.**

Forward and reverse primers were designed manually from an alignment of the coding sequences of IRACs. Each primer pair was designed to amplify a fragment from a specific Irac and generate an amplicon with a particular size.
